# Chemical Modification of Tiopronin for Dual Management of Cystinuria and Associated Bacterial Infections

**DOI:** 10.1021/acsami.3c07160

**Published:** 2023-09-06

**Authors:** Anil Kumar, Lori M. Estes Bright, Mark Richard Stephen Garren, James Manuel, Arpita Shome, Hitesh Handa

**Affiliations:** † School of Chemical Materials and Biomedical Engineering, University of Georgia, Athens, Georgia 30602, United States; ‡ Pharmaceutical and Biomedical Sciences Department, College of Pharmacy, University of Georgia, Athens, Georgia 30602, United States

**Keywords:** tiopronin-NO, dual management, cystinuria, antibacterial, biofilm, prevention

## Abstract

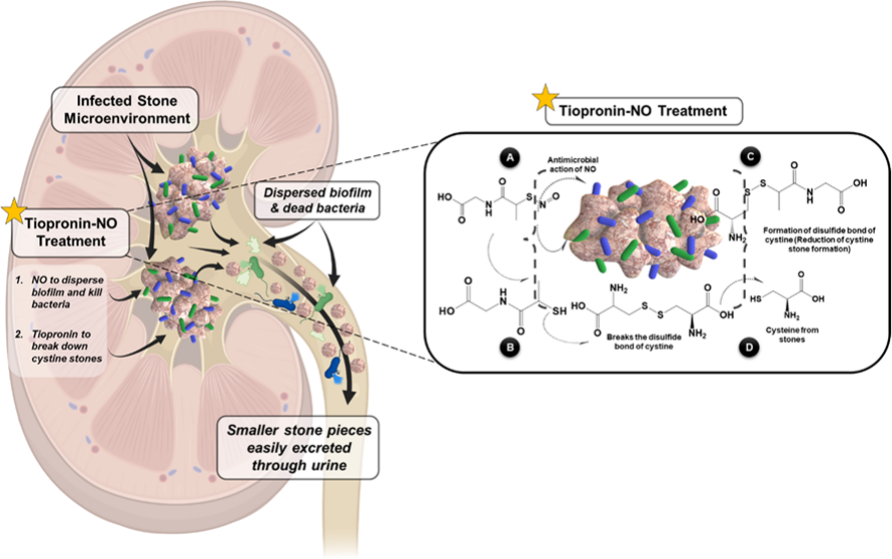

Cystinuria is an inherited autosomal recessive disease of the kidneys of recurring nature that contributes to frequent urinary tract infections due to bacterial growth and biofilm formation surrounding the stone microenvironment. In the past, commonly used strategies for managing cystinuria involved the use of (a) cystine crystal growth inhibitors such as l-cystine dimethyl ester and lipoic acid, and (b) thiol-based small molecules such as *N*-(2-mercaptopropionyl) glycine, commonly known as tiopronin, that reduce the formation of cystine crystals by reacting with excess cystine and generating more soluble disulfide compounds. However, there is a dearth of simplistic chemical approaches that have focused on the dual treatment of cystinuria and the associated microbial infections. This work strategically exploited a single chemical approach to develop a nitric oxide (NO)-releasing therapeutic compound, *S*-nitroso-2-mercaptopropionyl glycine (tiopronin-NO), for the dual management of cystine stone formation and the related bacterial infections. The results successfully demonstrated that (a) the antibacterial activity of NO rendered tiopronin-NO effective against the stone microenvironment inhabitants, *Escherichia coli* and *Pseudomonas aeruginosa*, and (b) tiopronin-NO retained the ability to undergo disulfide exchange with cystine while being reported to be safe against canine kidney and mouse fibroblast cells. Thus, the synthesis of such a facile molecule aimed at the dual management of cystinuria and related infections is unprecedented in the literature.

## Introduction

Cystinuria, a commonly occurring autosomal kidney stone disorder, leads to significant morbidity in affected patients due to the recurrent nature of the disease that affects the proximal renal tubule and gastrointestinal tract.^1^ Patients with this disorder are unable to reabsorb dibasic amino acids such as cystine, ornithine, and lysine from their urine.^2^ Lack of reabsorption of cystine by the proximal tubule results in urinary cystine concentrations above 250 mg/L, ultimately leading to cystine stone formation.^3^ In the past, commonly used strategies for managing cystinuria involved the use of (a) cystine crystal growth inhibitors such as l-cystine dimethyl ester^4,5^ and lipoic acids^6^ and (b) FDA-approved, thiol-based small-molecule drugs such as *N*-(2-mercaptopropionyl) glycine, commonly known as tiopronin.^7,8^ The dibasic amino acid cystine is formed from two cysteine monomers joined by a disulfide bond. Tiopronin reduces cystine crystal formation by reacting with excess cystine and generating more soluble disulfide compounds (tiopronin–cysteine complex). However, there are no curative treatments for cystinuria in the current scenario, and patients have a lifelong risk of stone formation, repeated surgery, and impaired renal function.

Another major challenge associated with this kidney stone disorder is the continuous growth of bacteria surrounding the stone microenvironment, leading to an escalated urinary tract infection rate.^9,10^ Studies have shown that bacteria significantly impact the development of struvite urinary stone formation due to the combination of minerals produced by bacteria.^11,12^*Escherichia coli* and *Pseudomonas aeruginosa* are the most predominant bacterial species isolated from stone cultures and involved in struvite stone formation.^13^ Some bacteria, such as *Staphylococcus aureus*, enter the kidneys through the bloodstream, while urinary tract infections caused by *Proteus mirabilis* usually occur in patients under long-term catheterization.^14,15^ Thus, the continuous growth of bacteria around the stone microenvironment severely increases the complexity of renal conditions and intensifies the challenges for treatment procedures. The use of antibiotics in treating clinical infections is the current standard of care, though many research and clinical professionals warn against the dangers of antibiotics.^16^ Misuse and overprescribing have led to the development of antibiotic-resistant microorganisms that can persist beyond antibiotic treatment, requiring higher doses and multiple antibiotic-cocktail trials to achieve infection relief.^17^ Moreover, bulky antibiotic molecules are unable to penetrate biofilm matrices, often present in stone infections.^18,19^ Therefore, broad-spectrum antimicrobials other than antibiotics must be explored as infection treatment options. Further, to date, kidney stone disorder remediation methods have focused on managing either cystinuria or stone-related bacterial infections. None of the approaches have been aimed toward the dual and simultaneous treatment of cystinuria and microbial infections surrounding the stone microenvironment.

Nitric oxide (NO), an endogenous gaseous signaling molecule, has been extensively explored as an alternative to antibiotics for the treatment of bacterial and viral infections.^20−24^ As NO is soluble in both water and lipids, it can freely diffuse across cellular membranes to initiate nitrosative and oxidative damage leading to microbial death.^25^ These imposed stresses are caused by the reactive nitrogen species (RNS) and reactive oxygen species (ROS) that are produced when the highly reactive NO interacts with O_2_ in the environment.^26,27^ Further, the gaseous nature of NO allows it to penetrate biofilm matrices, and several studies have shown its ability to actively disperse biofilms through disruption of intracellular second messenger cyclic di-GMP, inducing greater bacterial mobility and scattering.^28−30^ Over the years, NO-releasing, antifouling surfaces have been widely developed to combat medical device-associated microbial infections.^31−33^ The multimechanistic killing and biofilm dispersal capabilities of NO make it an effective alternative to antibiotics, especially in the case of urinary stones where bacterial biofilms render antibiotics obsolete.^34^*S*-Nitrosothiols are small-molecule drugs that contain NO bound to a thiol (sulfhydryl) group (R-SH) that has shown immense potential against common Gram-negative and Gram-positive bacterial infections.^35,36^ The NO release from such small molecules is due to the homolytic cleavage of the S–N bond in the physiological environment.^37,38^ The mechanism involves the process of transnitrosylation which facilitates the transfer of bound NO from *S*-nitrosothiols to other thiol groups, resulting in thiyl radical byproducts.^39,40^ Subsequently, these thiyl radicals can react with reduced thiols to form disulfide bonds in the physiological environment.^41^ Despite this, the potential of *S*-nitrosothiols has never been explored for the dual action and simultaneous treatment of bacteria-associated stone infections and cystinuria.

**Scheme 1:**
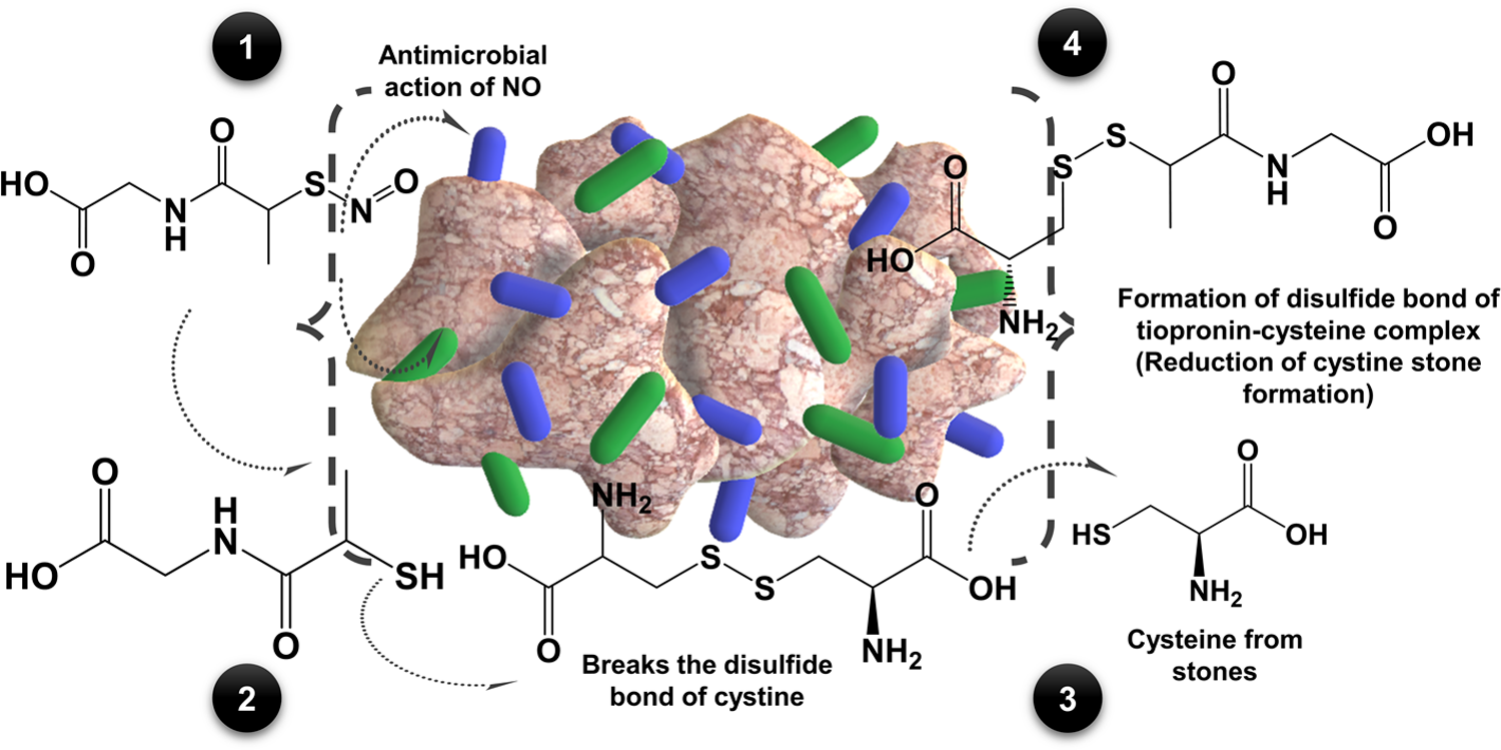
Infectious Cystine Stone Microenvironment and the Therapeutic Mechanism of Tiopronin-NO in the Inhibition of Bacterial Cell Growth and Reduction of Cystine Stones (1–4) (1) Tiopronin-NO effectively kills the bacteria due to the bactericidal nature of NO, causing oxidative stress and eventually damages the cellular machinery resulting in bacterial cell death. (2) The NO release from these small molecules is due to the homolytic cleavage of the S–N bond in the physiological environment. The mechanism involves the transfer of bound NO from *S*-nitrosothiols to other thiol groups through a process known as transnitrosylation and functioning as the parent molecule (tiopronin). (3) Tiopronin breaks the disulfide bonds of cystine and (4) attaches to cysteine to become a Tiopronin–cysteine complex, which dissolves more readily, disseminating cystine stones.

Therefore, we hypothesized that developing a NO-releasing small therapeutic molecule like *S*-nitroso-2-mercaptopropionyl glycine (tiopronin-NO) will provide a single, dual-action avenue in (a) reducing cystine stone formation and (b) treatment of bacterial infections near the stone microenvironment as shown in Scheme 1. To the best of our knowledge, this is the first report that exhibits the prominence of a single chemical approach to investigate the antimicrobial effects of tiopronin-NO and the ability of thiol drugs to undergo disulfide exchange with cystine for the treatment of cystinuria and infectious stone formation.

## Materials and Methods

### Materials

2-Mercaptopropionyl glycine (tiopronin), *tert*-butyl nitrite, l-cystine, sodium cyanide, sodium nitroprusside, and acetic acid were purchased from Sigma-Aldrich (St Louis, MO). Hydrochloric acid, sodium hydroxide, methanol, and NO labeling 4-amino-5-methylamino-2′,7′-difluorofluorescein diacetate (DAF-FM) dye and Filmtracer LIVE/DEADTM Biofilm viability kit were obtained from Thermo Fisher (Waltham, MA). The animal LIVE/DEAD cell staining kit was purchased from Biotium (Fremont, CA). Ultrapure Milli-Q water (resistivity of 18.2 MΩ·cm) was used to obtain deionized (DI) water for buffer and reagent preparation. Crystal violet was obtained from Fisher Scientific (Waltham, MA).

Luria–Bertani (LB) agar and broth were purchased from Difco Laboratories, Inc. (Detroit, MI). Tryptic soybean agar (TSA) and broth (TSB) were purchased from Sigma-Aldrich (St. Louis, MO). Phosphate-buffered saline (PBS, pH 7.4) containing 138 mM NaCl, 2.7 mM KCl, and 10 mM sodium phosphate was used for all *in vitro* experiments and was acquired through VWR (Radnor, PA). Dulbecco’s modified Eagle’s medium (DMEM) and trypsin-EDTA were purchased from Corning (Manassas, VA 20109). The Cell Counting Kit-8 (CCK-8) was obtained from Sigma-Aldrich (St. Louis, MO 63103). The antibiotic penicillin-streptomycin (Pen-Strep) and fetal bovine serum (FBS) were purchased from Gibco-Life Technologies (Grand Island, NY 14072). *Escherichia coli* (ATCC 25922TM, *E. coli*) and *Pseudomonas aeruginosa* (ATCC 9027TM, *P. aeruginosa*) were used for all bacterial and biofilm experimental assays. Mouse fibroblast cells (3T3) and canine kidney cells (HDMCK) were originally obtained from American Type Culture Collection (ATCC). All required chemicals were used without further purification unless otherwise noted.

### Methods

#### Synthesis of *S*-Nitroso-2-mercaptopropionyl Glycine (Tiopronin-NO)

Tiopronin-NO was synthesized as illustrated in Scheme S1, with the help of previously reported methods with slight modification.^42^ 2-Mercaptopropionyl glycine (tiopronin) was dissolved in Milli-Q water and stirred continuously at 0 °C using an ice bath for 30 min. Following this, 1 M HCl was added to an equimolar amount of tiopronin and the mixture was further stirred for another 30 min. Once the solution was mixed completely, 2 M chelated *tert*-butyl nitrite solution was added dropwise to the reaction mixture. The reaction mixture was left to stir for another 1 h in an ice bath system. A wine-red color of the solution was observed after the successful exchange of thiol to NO. The reaction mixture was transferred into a round-bottom flask, and the byproduct *tert*-butanol was evaporated using a rotavapor at 50 °C. Finally, the compound was lyophilized and dried under vacuum, and the dry red powder of tiopronin-NO was collected and stored at 20 °C in dark conditions. The yield of the final product was found to be 95.0 ± 0.2%.

#### Characterizations of *S*-Nitroso-2-mercaptopropionyl Glycine (Tiopronin-NO)

Successful nitrosation of the thiol groups of 2-mercaptopropionyl glycine was confirmed using nuclear magnetic resonance spectroscopy (NMR). Samples were prepared by dissolving ∼10 mg of the sample in 1 mL of deuterated water (D_2_O) at room temperature. ^1^H NMR analysis was performed using a Varian Mercury 300 MHz NMR spectrometer in deuterated water with a total of 256 scans set up. Chemical shifts were reported in parts per million (ppm). High-resolution mass spectrometry (HRMS) is an analytical technique used to measure the mass-to-charge ratio of ions. The analysis was performed using the Orbitrap Elite system (Thermo Scientific), and the samples were injected in methanol. For UV–visible spectroscopy, the samples were dissolved in water and the spectra were collected on a scan range of 200–700 nm at 37 °C by using a Cary 60 UV–vis spectrophotometer.

Fourier transform infrared (FTIR) spectroscopy was performed using a Spectrum Two Fourier transform infrared spectrometer from PerkinElmer using the KBr loading method. In brief, lyophilized samples of tiopronin and tiopronin-NO were mixed with anhydrous KBr to achieve a final weight percent of 1%. The mixed powder (100 mg) was loaded into a 7 mm diameter die and pressed with an applied force of 1.5 tons for 5 min (Piketech). Following a baseline reading, the pressed KBr disk was read using a scan range of 4000 to 650 cm^–1^ with a resolution of 4 cm^–1^. A total of 32 scans were performed per run with each sample prepared in triplicate. Each measurement was baseline-corrected for analysis.

Nitric oxide release from tiopronin-NO was characterized using Sievers Chemiluminescence NO Analyzers (NOA 280i, GE Analytical, Boulder, CO). Chemiluminescence detection methods utilize the NO reaction with ozone, as shown below, to detect the emitted photon from the excitation reaction.
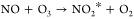

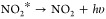


The voltage signal is multiplied and then converted to a parts per billion or parts per million measurement that is displayed on the NOA screen. Using the raw data in ppb/ppm form and an NOA constant (mol ppb^–1^ s^–1^), the data is normalized for the moles of tiopronin-NO in the solution being tested and converted to an NO release unit (×10^–10^ mol NO min^–1^ mol tiopronin^–1^). The sample chamber was maintained at 37 °C using a water bath and an amber vial, ensuring NO release was not catalyzed by any outside light source. Each sample was prepared at 2× concentration and stored at −80 °C until measurements were taken. Before sample measurement, a PBS solution was adjusted to the ideal pH (5.5, 6.5, and 7.4), and 1 mL was added to the sample chamber. Once a baseline reading was recorded, 1 mL of the preprepared 2× concentrated tiopronin solution was injected into the reaction chamber, diluting to the final concentrations of 5, 10, and 20 mM. Three replicates were measured for each concentration.

#### Antibacterial Activity of Tiopronin-NO

The antibacterial activity of tiopronin and tiopronin-NO was investigated based on a modified version of the American Society for Testing and Materials E2180 protocol. *E. coli* and *P. aeruginosa* were used for antimicrobial evaluation as they are the most common bacterial species isolated from urinary stone cultures.^13^ Bacteria were cultured in an LB (*E. coli*) or TSB (*P. aeruginosa*) broth at 37 °C and 150 rpm until the bacterial log phase was reached. The resulting culture was collected by centrifugation, rinsed with PBS, and resuspended in PBS for exposure to the treatment solution. In a 48-well plate, 200 μL of tiopronin or tiopronin-NO (20, 10, 5, and 2.5 mM) and 800 μL of 0.1 OD bacteria (∼10^8^ CFUs) were added to individual wells. The well plate was kept at 37 °C and 150 rpm for 24 h. Following 24 h of incubation, 100 μL was removed from each well, serial dilutions were performed, and several chosen dilutions were plated on TSA or LB agar plates. Viable CFUs were counted after agar plates were incubated at 37 °C for 18 h.

#### Antibacterial Activity of Tiopronin-NO Using Flow Cytometry

Bacterial cultures used for flow cytometry were prepared based on the instructions provided with the Viability/Cytotoxicity Assay for Bacteria Live & Dead Cells (Biotium, Catalog Number: 30027). A turbid overnight culture of *E. coli* (LB) and *Pseudomonas* (TSB) was subcultured at a 1:20 dilution in fresh media and grown until an OD_600_ of 0.4–0.6 was reached. They were then seeded into a six-well plate, treated with 20 mM of the drug solution, and incubated for 24 h at 37 °C in an orbital shaker (150 rpm). The cells were harvested by centrifugation at 7000*g* for 10 min at room temperature, washed with PBS (1×), and concentrated 10-fold in the same solution.

Flow cytometry experiments were performed using a Bachmann instrument from BD Biosciences (San Jose, CA), which is equipped with 3 lasers (violet, 405 nm; blue, 488; and red, 640 nm) as well as forward and side scatter detectors. Channels 530/30 and 695/40 were used for DMAO and EthD-III detection, respectively. The Live/Dead assay kit is composed of two fluorescent dyes, DMAO and EthD-III. DMAO penetrates cell membranes and can complex with bacterial DNA. In contrast, EthD-III is able to interact with bacterial DNA only if the lipid bilayer is damaged, indicating that EthD-III bacteria are ruptured or dead. Briefly, 1.5 μL of both DMAO and EthD-III was added to each 100 μL of bacterial suspension containing 10^8^ CFU/mL. Samples were vortexed gently and incubated at room temperature in the dark for 15 min. The laser calibrations with optimized filters for the detection of fluorescence were selected and scatter plotted as such: Q1 (red) and Q4 (green) are cells strongly expressing one and only one color (single positives), Q2 dissects cells with both red and green positivity (double positives), and Q3 is cells negative for both colors. The data analysis was performed through FlowJo software.^43,44^

#### Intracellular Detection of ROS/NO Species in Bacterial Cells

The detection of the uptake of NO into the cellular system was performed as reported by Kumar et al. with modification for application to a bacterial study.^45^ The bacteria were cultured in LB (*E. coli*) or TSB (*P. aeruginosa*) broth at 37 °C and grown in a chamber slide (Nunc Lab Tek, Thermo Fisher Scientific) inoculated with 200 μL of bacterial cell suspension. Then, the bacteria were treated with 200 μL (10 mM) of tiopronin or tiopronin-NO in a bacterial growth medium. Following 24 h of incubation, the bacterial cells were stained with nitric oxide labeling dye, 4-amino-5-methylamino-2′,7′-difluorofluorescein diacetate (DFM-FM, 5 μM), and DNA labeling dye, EthD-III (5 μM), for 30 min at 37 °C. Control groups were not treated with tiopronin or tiopronin-NO but were stained with labeling dyes. To remove free dye molecules, the cells were washed with saline three times and imaged under a confocal laser microscope (Zeiss, LSM 710) with the following setup: DAF-FM, Ex/Em = 495/515 nm; and EthD-III ex/em = 553/568 nm. Images were captured using Zen system software (Carl Zeiss Canada Ltd., Toronto, ON, Canada).

#### Biofilm Dispersion Assay

Biofilms were grown in chamber slides as described previously, with slight modifications.^46^ Briefly, chamber wells (Nunc Lab-Tek Chamber Slide, Thermo Fisher) were sterilized with a 10% bleach solution and rinsed with sterile water, followed by UV radiation for 1 h. The overnight culture of bacteria grown in LB or TSB was diluted to an optical density (OD_600_) of 0.005 in the appropriate media, and cells were grown for another 2–3 h. Further, the cultures were diluted with a 1:2000 ratio and inoculated with 200 μL of bacterial cell suspension. Every 8 h the medium was replaced with prewarmed fresh medium and allowed to grow for 24 h. The drug concentration of 20 mM was then provided to each treatment group except the controls. For visualization, we used a FilmTracer, (LIVE/DEAD) biofilm viability analysis dye, which stains the live (green) and dead (red) cells within the biofilm, based on the membrane integrity. The biofilms attached to the bottom surface of the plates were stained with 1.5 μl of DMAO and 3 μl of the-III for 30 min at 37 °C. Confocal microscopy was performed using a Zeiss LSM 700 microscope, and images were captured using the Zen system software (Carl Zeiss Canada Ltd., Toronto, ON, Canada).

#### Microtiter Plate Assay for Biofilm Quantification

The biofilms of *E. coli* and *P. aeruginosa* were grown on pre sterilized 96-well flat-bottom polystyrene microtiter plates in triplicate as described previously.^47^ In brief, the overnight cultures of bacteria were diluted to an optical density at 600 nm (OD_600_) of 0.005 in the appropriate medium, and cells were grown for another 2–3 h. Further, the cultures were diluted at a 1:2000 ratio and inoculated with 200 μL of bacterial cell suspension. Next, 200 μL of sterile PBS was added to peripheral wells to reduce the water loss. The plates were incubated for 24 h at 37 °C to allow biofilm growth. Every 8 h, the medium was replaced with prewarmed fresh medium without disturbing the monolayer of the culture. The biofilms were treated with 20 mM of each drug (except the control) and further incubated for 24 h. After treatment, biofilms were fixed with 100% methanol, washed twice with PBS (1×), and air-dried. Then, 200 μL of sterile crystal violet solution (0.2%) was added to all wells. After 15 min, the excess crystal violet was removed and plates were washed twice with PBS and air-dried. Finally, the cells stained with crystal violet were dissolved in 125 μL of 33% acetic acid prepared in an aqueous solution. The growth of the biofilm was measured in terms of OD_590_ using a microplate reader (Biotek, Winooski, VT). Three independent experiments were performed, and the results were analyzed.

#### Biofilm Biomass Quantification

For biomass analysis of the biofilm matrix, biofilms were grown in six-well polystyrene microtiter plates for 24 h and treated with 20 mM of tiopronin or tiopronin-NO, similarly used for CV quantification. After 48 h, the biofilm matrix was extracted using a slight modification to a previously described protocol.^48^ Briefly, biofilm samples were scraped from the six-well plates, resuspended with ultrapure water, sonicated (Ultrasonic Processor, Fisher) for 30 min in a water bath, and then vortexed for 3 min. The suspension was centrifuged at 13 000 rpm for 15 min at 4 °C, and the cell pellets were dried at 90 °C until a constant dry biofilm weight was determined.

#### Scanning Electron Microscopy (SEM) of Treated Bacterial Biofilms

The biofilms were grown in a 24-well plate and treated with the same concentrations of the drug used for the biofilm quantification study. At 48 h, the medium was aspirated, and nonadherent cells were removed by washing using saline (0.9% NaCl). The cells were fixed with 30% glutaraldehyde for 24 h at 4 °C, washed with saline three times, and then dehydrated for 10 min in 30% ethanol, 10 min in 50% ethanol, 10 min in 70% ethanol, 10 min in 80% ethanol, 10 min in 90% ethanol, and 10 min in 100% ethanol. They were then soaked in 100% ethanol twice for 15 min each. The samples were immediately transferred to a 2:1 ratio of 100% ethanol and HMDS and then to a 1:2 ratio of 100% ethanol and HMDS. Before observation, the bases of the wells were mounted onto aluminum stub holders, sputter-coated with gold–palladium, and observed with an FEI Teneo Scanning Electron Microscope (Thermo Fisher, Waltham, MA).

#### Urine Sample Preparation

The urine sample was obtained from Discovery Life Sciences (Huntsville, AL) and work was performed according to the Use of Laboratory Sample Guidelines by the University of Georgia to ensure appropriate handling and disposal of clinical samples. The artificial urine was used to investigate the effects of the drug on cystine solubility at different pHs. To measure the cystine solubility in the patient urine sample, the experiment was prepared according to a previously reported protocol with slight modifications.^49^ Briefly, 0.9 mL of urine sample was added to tubes containing 1 mg of cystine, and 0.1 mL of tiopronin or tiopronin-NO solution was then added to bring all samples to a final volume of 1 mL. The stock solution (20 mM) of tiopronin or tiopronin-NO added to the tubes brought the final drug concentration in urine aliquots to 2 mM. 1 mL of clinical laboratory reagent water was used as a control group throughout the experiment. The concentration was selected based on the clinical setting to analyze the interaction of the drug in urine samples by the Mayo Clinic Rental Testing Laboratory, Rochester, MN.

The urine samples were incubated at 37 °C with constant stirring for different time points (0, 5, 1, 3, 12, 24, and 48 h). After incubation, the samples were centrifuged at 3000 rpm for 20 min to harvest the remaining solid-phase cystine. The supernatant was discarded, and the remaining pellet was dissolved in a high-pH buffer composed of 10 mL of 0.1 M sodium carbonate (pH 9.9). Finally, the cystine concentration was measured and compared to the amount of cystine in the tube before the urine sample was added to determine the amount of cystine dissolved in the urine sample. Three independent assays were run separately and averaged to analyze the solubility of cystine in clinical urine samples. The coefficient of variation for the cystine solubility assay was 3.8%.

#### Cystine Solubility (Sodium Nitroprusside Assay)

The cystine solubility was measured according to the previous assay reported by Nakagawa et al.^50^ and Goldfarb et al. with slight modification,^51^ including designing the assay to be completely run in Coring Costar microplates (96-well, clear flat-bottom, UV transparent, polystyrene). Briefly, 50 μL of a urine sample (including control in a separate well), 90 μL of 0.1 M phosphate buffer (pH 7.4), and 30 μL of 10% (w/v) sodium cyanide solution (prepared in fume hood) were added to the plate and incubated at room temperature with constant shaking for 20 min. Next, 20 μL of 10% (w/v) of sodium nitroprusside was added, and the samples were incubated for 3 min at room temperature with constant shaking. After a short incubation period of 3 min, the absorbance of the cysteine–nitroprusside complex was measured immediately at 521 nm using a plate reader. This assay was performed as three independent experiments with a total of *n* = 5 replicates.

#### Mass Spectroscopy Measurement of Drug Complex

Tiopronin and tiopronin-NO samples were analyzed by LC-MS at the Proteomics and Mass Spectrometry Facility (PMSC), Department of Chemistry, University of Georgia. Samples were injected, drugs were separated using a C18 column, and the full range of drug peaks and possible drug complex formation peaks were targeted and analyzed.

#### Biocompatibility Study

Mouse fibroblast (3T3) and canine kidney cells (HDMCK) were cultured in 25 cm^2^*T*-flasks containing DMEM supplemented with 10% fetal bovine serum (FBS) and 1% penicillin-streptomycin to avoid any contamination. The cells were incubated at 37 °C in a 5% CO_2_ humidified environment to allow for monolayer formation. The culture medium was replaced every 2 days, and the cells were allowed to grow to confluency above 80%. After that, the cells were trypsinized (0.18% trypsin and 5 mM EDTA) and seeded at a density of 5 × 10^4^ cells/well into 96-well plates, and plates were further incubated at 37 °C in a 5% CO_2_ humidified environment. The cells were treated with various concentrations of tiopronin or tiopronin-NO at 24 and 72 h, and the effects of the drugs on cell viability were determined by a CCK-8 cell counting kit according to the manufacturer’s protocol (Sigma-Aldrich). The absorbance was detected at 450 nm. This assay was performed as three independent experiments with a total of *n* = 5 replicates. The data were expressed as mean ± standard deviation (SD), and the cell viability was calculated using the following equation:^8^
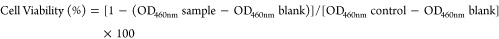
where “sample” and “control” mean the cells with and without treatment with tiopronin and tiopronin-NO, respectively.

#### Confocal Microscopy Analysis (Live and Dead Assay)

Mouse fibroblast (3T3) and canine kidney cells (HDMCK) were grown in a chamber slide with a cell density of 3 × 10^5^ and treated with 20 mM tiopronin or tiopronin-NO for 24 h. The effects of the drugs in terms of cell viability were determined with Live/Dead staining (Live/Dead BacLight bacterial viability assay kit, L13152, Invitrogen, Eugene), according to the manufacturer’s protocol. In brief, 2 μM SYTO 9 (green) and 4 μM propidium iodide-PI (red) were added to each well and incubated at room temperature for 20 min. The stained cells were observed using confocal microscopy using a Zeiss LSM 700 microscope and images were captured using the Zen system software (Carl Zeiss Canada Ltd., Toronto, ON, Canada). For each treatment group, the fluorescence intensity was measured at emission wavelengths of 530 nm (green) and 620 nm (red).

### Statistical Analysis

All data are expressed as mean ± standard deviation (SD) of the triplicate experimental data. A two-tailed Student *t* test was used to determine the significance of differences in biofilm formation between the control and each group. The *p*-value < 0.05 was taken as significant. Linear regression was used to determine the correlation of solubility with pH, and the Spearman rank correlation was used to determine increases in solubility with time with *p* < 0.05 considered significant.

## Results and Discussion

### Synthesis and Characterization of *S*-Nitroso-2-mercaptopropionyl glycine

The therapeutic mechanism of tiopronin-NO involves the release of NO from the molecule under physiological conditions, allowing for bacterial killing within the stone microenvironment. Subsequently, the tiopronin parent molecule breaks the disulfide bond of cystine within the stone and attaches to free cysteine via a disulfide bond for further dissolution and dissemination from the body (Scheme 1). This novel dual-action system tackles both the microbial burden and the stone degradation pathway in a single treatment. Scheme S1 illustrates the synthesis of the novel NO-coupled small-molecule compound *S*-nitroso-2-mercaptopropionyl glycine (tiopronin-NO) following the *S*-nitrosation of the thiol precursor 2-mercaptopropionyl glycine (tiopronin) using *tert*-butyl nitrite (TBN). *tert*-Butanol is the only reaction byproduct, thus classifying TBN as a green reagent that has previously been reported for the *S*-nitrosation of thiols and other reactive groups such as phenols, alkenes, alkynes, acetanilides, and sulfonamides.^52^

Chemical modification of the thiol group to *S*-nitroso was confirmed by the absence of a thiol proton in comparison with the NMR spectra of 2-mercaptopropionyl glycine, as shown in Figure S1. The signal at 3.55 ppm corresponds to the proton adjacent to the SH group, which is a multiplet resulting from the complex *J*-coupling of 3 protons on the adjacent carbon atom and the proton on the sulfur (i.e., a doublet of quartets). After the nitrosation reaction, the resultant multiplet signal at 3.55 ppm changed to a single quartet, showing the proton loss from the adjacent sulfur atom. This was further evidenced by the loss of associated peaks from the proton on the reduced thiol upfield (Figure S1). The evidence of sulfur proton removal in the NMR spectra and confirmation of NO release from the compound from NOA analysis indicates the presence of an *S*-nitrosothiol group with the corresponding structure. Further, high-resolution mass spectrometry (HRMS) was performed to confirm the expected molecular weight of the compound. HRMS analysis validated the successful nitrosation of 2-mercaptopropionyl glycine through peaks corresponding to the deprotonated conjugates in negative-ion mode operation (Figure S2). From the calculated theoretical isotope distribution of tiopronin (molecular formula: C_5_H_8_N_2_O_4_S = 192.19), a characteristic product ion of *m*/*z* 191.01 corresponding to deprotonated glycine was recorded in rich abundance for most of the glycine conjugates.

Moreover, tiopronin-NO was further subjected to ultraviolet–visible (UV–vis) spectral analysis to demonstrate the successful nitrosation of 2-mercaptopropionyl glycine. A characteristic peak in the 320–430 nm region (Figure S3a) was observed that was attributed to the ππ* transition of –N=O, thus validating the nitrosation step. Furthermore, Fourier transform infrared spectroscopy was performed to ascertain the successful nitrosation, as shown in Figure S3b and c. Tiopronin-NO exhibited broadened stretching vibrations around the amide II band (ν_NO_ = 1537 cm^–1^) indicative of nitrosothiol formation. The emergence of a broad band at a low wavenumber (ν = 650–500 cm^–1^) provided further evidence of disulfide bridge formation, a byproduct of the homolytic cleavage of the *S*-nitrosothiol bond in the presence of light, heat, and metal ions. Further analysis showed several peaks characteristic of tiopronin in agreement with previous literature (Figure S3b),^53^ with confirmation of the nitrosation of the compound.

**Figure 1:**
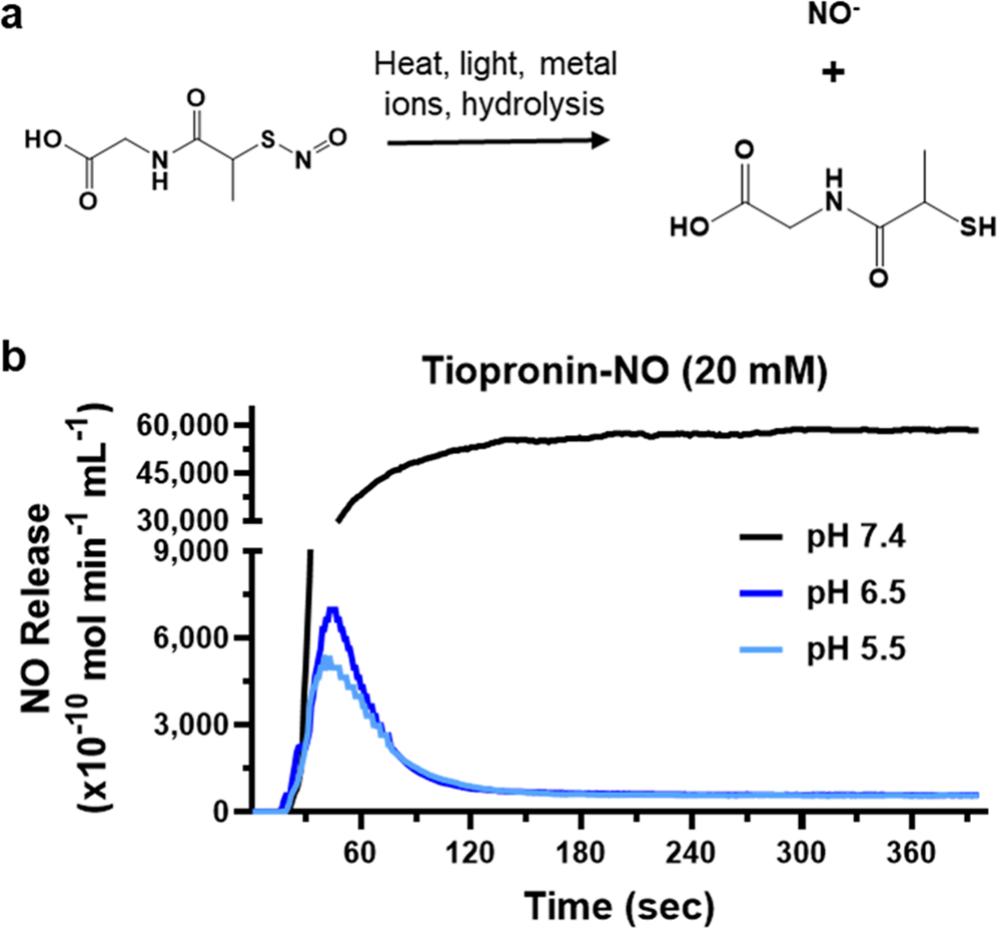
NO release characteristics from tiopronin-NO. (a) Release of NO from tiopronin-NO that occurs in physiological conditions. (b) NO release from 20 mM tiopronin-NO solution tested at pH of 5.5, 6.5, and 7.4 using a nitric oxide analyzer.

The characterization of NO release from tiopronin-NO was performed using chemiluminescent detection methods considered to be the gold standard for NO release measurements. Tiopronin-NO at a concentration of 20 mM was tested, corresponding to the concentration used for cystine treatment and antibacterial tests. Release of NO was characterized at pH levels of 5.5, 6.5, and 7.4 to examine NO release, in accordance with the tiopronin-cystine activity assay. In clinical settings, alkalinization (pH 7.5–8.0) is essential to enhance cystine solubility in the urine.^54−56^ Hence, we selected different pH concentrations to evaluate the potential therapeutic activity of the synthesized dual-action compound and its NO-releasing ability under physiological conditions (37 °C and shielded from light; Figure 1a). Critically, *S*-nitrosothiol compounds like tiopronin-NO are known to exhibit environment-specific decomposition kinetics, dependent on pH, buffer concentration, temperature, and the presence of various components such as reduced thiols, metal ions, and proteins.^57,58^

The drug performed similarly at pH 5.5 and 6.5 with an abrupt burst release within the first 90 s, followed by a slow, stabilized release of 6.12 ± 0.66 and 6.29 ± 1.26 (×10^–10^ mol NO min^–1^ mL^–1^), respectively (Figure 1b). However, at pH 7.4, tiopronin-NO released NO at a level of 741.14 ± 8.92 (×10^–10^ mol NO min^–1^ mL^–1^) for ∼20 min followed by a stabilized continuous release of NO at 0.21 ± 0.12 (×10^–10^ mol NO min^–1^ mL^–1^). As previously reported, the enhanced release of NO at pH 7.4 was attributed to the pH-dependent stability of RSNO molecules.^59^ In short, the stability of RSNOs such as *S*-nitrosoglutathione (GSNO) and *S*-nitroso-*N*-acetylpenicillamine (SNAP) can be improved in acidic conditions (pH ∼ 5) due to the protonation of the RSNO oxygen, strengthening the S–N bond.^59,60^ Tiopronin-NO fabricated in this study was observed to have similar pH-dependent stability, displaying higher levels of NO release due to increased homolytic cleavage of the nitrosothiol bond at pH 7.4. Thus, the pH-dependent stability with enhanced NO release is beneficial for penetrating preformed biofilms and eradicating infectious pathogens.

### *In Vitro* Antimicrobial Activity

To investigate the antimicrobial activities of tiopronin-NO, the most common bacterial species found around the stone environment were utilized, i.e., *E. coli* and *P. aeruginosa*.^13,61^ For this study, we selected a maximum dose of 20 mM, 250–500 times lower than the clinical therapeutic dose^62^ (6–12 molar or ∼1000–2000 mg/day) to understand the antibacterial efficacy of tiopronin and tiopronin-NO.

**Figure 2:**
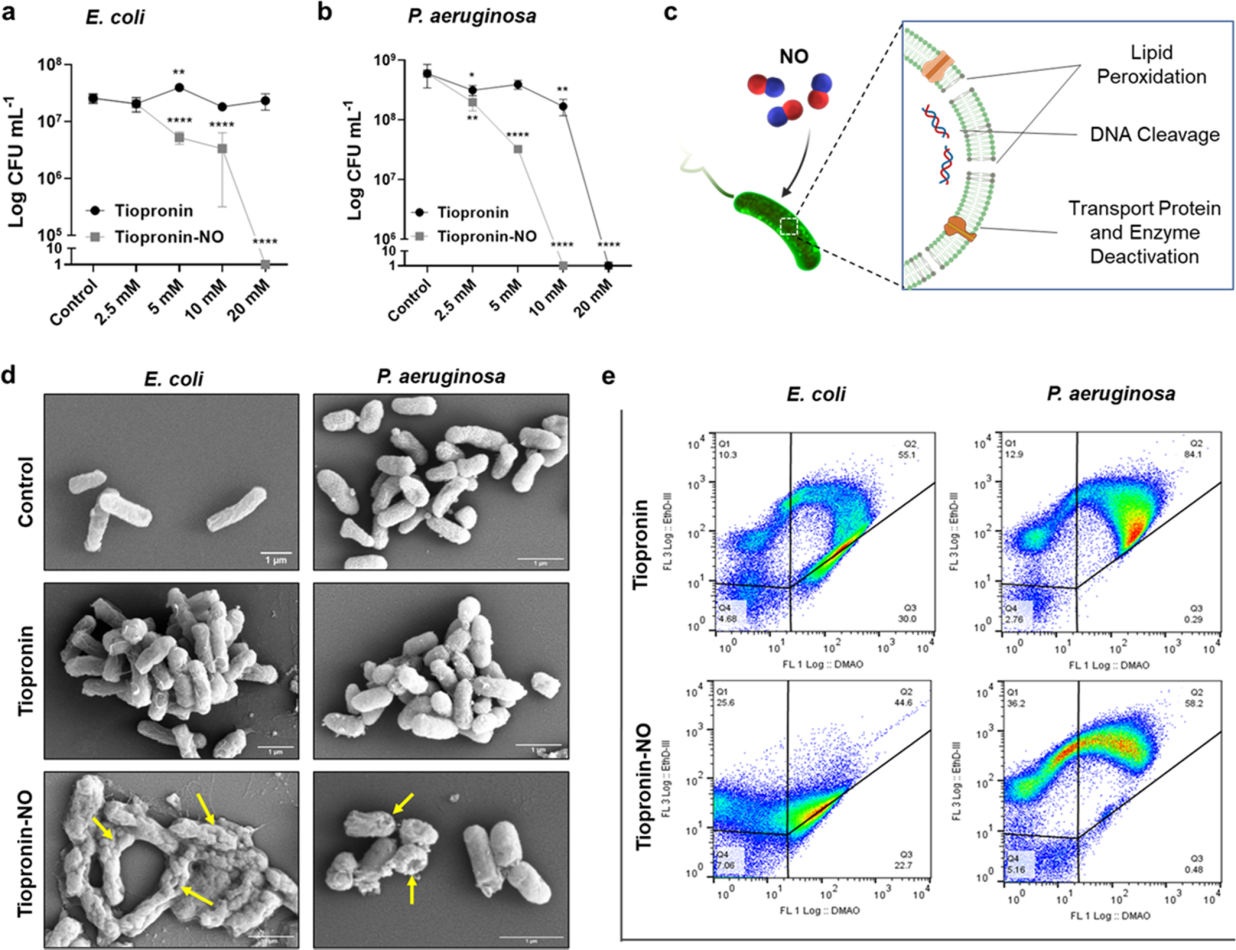
Antimicrobial efficacy of tiopronin-NO against *E. coli* and *P. aeruginosa*. (a) CFU reduction of *E. coli* and (b) *P. aeruginosa* after treatment with tiopronin and tiopronin-NO at several concentrations. (c) Antimicrobial mechanism of NO. (d) SEM images of untreated bacteria as well as bacteria treated with tiopronin and tiopronin-NO. Microbial membrane damage is denoted by yellow arrows. The scale bar represents 1 μm. (e) Flow cytometry analysis of dead bacteria after drug treatment. Q1 represents bacterial cells expressing EthD-III (dead), Q2 displays cells expressing both DMAO and EthD-III (alive but not entirely healthy), Q3 represents bacterial cells that are negative for both dyes, and Q4 shows bacterial cells only expressing DMAO (alive and healthy). Statistical significance is denoted as *p < 0.05; **p < 0.01; ****p < 0.0001.

There was a dose-dependent sensitivity to tiopronin-NO in the case of both *E. coli* and *P. aeruginosa*. The bacterial reduction against *E. coli* was 21.76, 79.96, 87.28, and 100% when exposed to 2.5, 5, 10, and 20 mM dosages of tiopronin-NO, respectively (Figure 2a). Tiopronin-NO also effectively killed *P. aeruginosa*, with reductions of 66.73 and 94.57% after exposure to 2.5 and 5 mM (Figure 2b). The complete killing of *P. aeruginosa* was achieved with treatments ≥10 mM for tiopronin-NO. The antibacterial effect of tiopronin is likely due to the thiyl radicals and ROS formed during the redox cycling between the thiol and the corresponding disulfide.^63^ Much like the ROS and RNS formed from NO’s reaction with environmental O_2_, high nitrosative and oxidative stress levels cause bacterial membrane and DNA damage and eventually cell death (Figure 2c). Following treatment with tiopronin-NO, similar thiyl radical formation and redox shuffling are expected due to homolytic cleavage of the *S*-nitrosothiol group. Moreover, the elevated levels of NO release can lead to antibacterial effects through the formation of ROS and RNS, which can cause lipid peroxidation, rupturing the bacterial membrane and damaging bacterial DNA, DNA repair systems, transport proteins, and intercellular mechanisms, thus preventing the bacteria from healing and surviving.^27,64,65^ This broad range of effects caused by NO and its reactive counterparts is due to nonspecific targeting, interacting with thiols, metal centers, and DNA, as well as inactivating metalloproteins and vital enzymes.^66^ The scanning electron microscopy (SEM) images of bacteria treated with 20 mM tiopronin-NO demonstrate the membrane-rupturing capabilities of NO, while tiopronin-treated bacteria appear similar to healthy untreated bacteria (Figure 2d).

Flow cytometry was employed to further confirm the above study and assess the therapeutic effects of tiopronin and tiopronin-NO on bacterial cells. A bacterial cell viability staining kit was used to determine the viability of individual cells, with green-emitting fluorescent DAMO stain binding to genetic material in living or dead cells and red-emitting fluorescent Ethidium Homodimer (EthD-III) only able to stain dead cells. Plotting the fluorescence signal intensity of FL3 (EthD-III-red) against FL1 (DMAO-green) after cell population gating enabled the quantification of the live/dead viability of bacterial suspensions following treatment with 20 mM of the drug concentration for 24 h.

In agreement with the CFU assay, we observed that both drugs demonstrated sensitivity in killing bacterial cells; however, tiopronin-NO displayed a greater sensitivity than tiopronin due to the presence of NO. The plots of FL3-(EthD-III) vs FL1-(DMAO) fluorescence intensity of treated bacteria are shown in Figure 2e, where the fluorescence events are divided into four regions based on their intensity in the two parameters as reported previously.^67^ Cells taking up (EthD-III) but weak in DMAO are assumed to be dead, while those that exclude EthD-III and retain DMAO are assumed to be alive.

The percentage of deceased bacteria in *E. coli* was found to be approximately 2.5 times higher in Tiopronin-NO treated samples compared to those treated with tiopronin alone. Similarly, *P. aeruginosa* exhibited a 3-fold increase in the same parameter. These findings are in agreement with the data obtained from colony-forming unit assays and further support the antibacterial capabilities of NO through various mechanisms.

### Intracellular Detection of ROS/NO Species in Bacterial Cells

The pathway of NO penetration into the cells to increase the bactericidal efficacy was illustrated using 4-amino-5-methylamino-2′,7′-difluorofluorescein diacetate (DAF-FM) as an NO-specific fluorescence probe. DAF-FM can penetrate the bacterial membrane efficiently and, upon hydrolysis of the acetate groups in the presence of intracellular esterases, forms membrane-impenetrable DAF-2. In the presence of NO, DAF-FM is nitrosated by reactive nitrogen species (e.g., N_2_O_3_) and emits a bright green fluorescence.^68^ Simultaneously, the bacterial cell labeled with EthD-III dye, a nucleic-acid-sensitive dye, emits bright red fluorescence upon interaction with genetic material (nucleic acids). The EthD-III is only able to enter cells with compromised cell membranes; therefore, the observation of red fluorescence inside the cells indicates the disruption of the plasma membrane, signaling cell death.

The increase in DAF-FM fluorescence intensity and the simultaneous increase in red fluorescence in tiopronin-NO-treated bacteria suggests the NO localization into the cellular compartment, thus damaging the cells and allowing EthD-III to enter both strains of bacteria (Figure S4a,b). Approximately 6.32-fold enhancement of green fluorescence signal and an 8.2-fold increase in red fluorescence signal were observed with tiopronin-NO treated *E. coli* (Figure S4c,d). Similarly, an 11.23-fold increase in green fluorescence and a 10.23-fold increase in red fluorescence were observed in the case of tiopronin-NO treatment of *P. aeruginosa* (Figure S4e,f). This result suggests that tiopronin-NO showed higher efficacy in killing the bacteria as compared with the untreated controls due to the high levels of oxidative stress and membrane degradation inflicted on bacteria by released NO.

### Disruption of Biofilm Formation and Analysis

**Figure 3:**
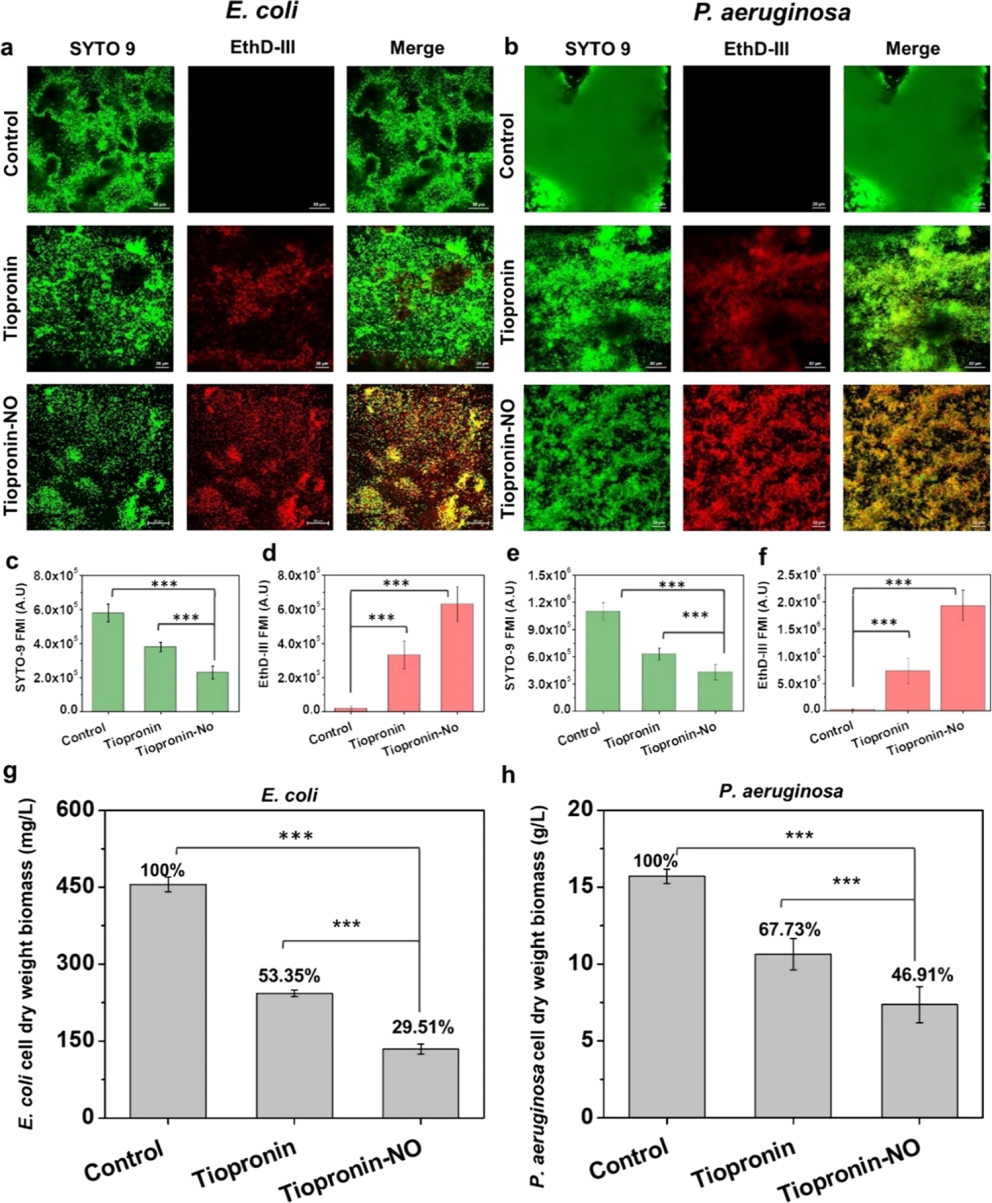
Biofilm disruption. (a) Images of *E. coli* and (b) *P. aeruginosa* biofilms stained with FilmTracer (Live/Dead) and imaged using confocal laser scanning microscopy after treatment with tiopronin and tiopronin-NO for 24 h (scale bar represents 5 μm). (c–f) Median fluorescence intensity of Syto-9 and Ethidium Homodimer III (EthD-III) based on imaging results from (a) and (b). (g, h) The dry weight of the biomass was measured after treatment with tiopronin and tiopronin-NO for 24 h. The values indicated are the average of three independent experiments. The results are shown as mean ± SD, and the asterisks represent a significant difference. *p < 0.05; **p < 0.01; ***p < 0.001; ns, no significant difference.

The formation and proliferation of organized bacterial communities on solid surfaces driven by environmental factors, called biofilms, is a naturally occurring phenomenon in bacterial physiology.^69^ These biofilms consist of accumulations of extracellular polymeric substance matrix (EPS) containing polysaccharides, proteins, glycolipids, and DNA of bacterial cells. The EPS layer shields the bacteria by preventing the penetration of drugs and macromolecules to reach the therapeutic site and diffuse through the biofilm matrix.^70^ Therefore, we evaluated the therapeutic effects of tiopronin-NO for the dispersion of preformed bacterial biofilms. To visualize the biofilm architecture and the dispersion response, biofilms were grown in chamber plates^71^ and then treated with 20 mM drug concentration for 24 h, similar to the CFU study. Further confocal imaging analysis was performed to understand the NO-induced dispersion of bacterial colonies within the biofilm formation.^72^ It was found that NO released from tiopronin-NO inhibited further colonization of bacteria and dispersed the already-formed biofilms. Dense colonies of the bacteria were scattered, and viable cell counts decreased while dead cell counts increased in both strains compared with untreated groups (Figure 3). Fluorescence analysis (Figure 3c–f) demonstrated the enhancement of the red signal in the case of tiopronin-NO treated groups compared to controls. The three-dimensional (3D) image analysis of the biofilms also confirms that tiopronin-NO causes significant damage to bacterial cell colonization compared to tiopronin and untreated controls (Figure S5). Significant enhancement of red signal was observed with the tiopronin-NO treated groups, thus clearly indicating that NO causes significant damage to bacterial cells, inhibiting further colonization and dispersing the preformed biofilms.

In addition, the SEM analysis of the biofilms shows that tiopronin-NO induced significant damage to the cell membranes within the biofilm matrix due to the therapeutic action of NO (Figure S6). Furthermore, the disruption and fracture of the bacterial cell membranes led to the deposition of internal cellular components throughout the biofilm structure. Thus, we conclude that NO interaction in the bacterial biofilm environment leads to membrane rupture of bacteria and dispersal of the preformed biofilms, preventing further colonization that leads to infection.

Finally, using dry-weight biomass analysis, the bactericidal properties in inhibiting biofilm formation were assessed against both drugs. As shown in Figure 3g,h, significant dry biomass weight reductions were observed for both treatment groups at a concentration of 20 mM compared to that of untreated bacteria. Therefore, we conclude that after modification of tiopronin with NO, significant enhancement of killing of bacterial cells as well as inhibition of biofilm formation due to the therapeutic nature of NO that halts bacterial growth and inhibits the proliferation of biofilms was achieved.

We performed quantitative analysis on biofilm formation and degradation using the standard crystal violet staining to further confirm this result.^73,74^ The dye colors the polysaccharide matrix and can be measured by optical absorbance at 590 nm, where absorbance readings are directly proportional to the amount of biofilm within the treated group. After staining, tiopronin-NO was found to be able to induce the dispersal of *E. coli* and *P. aeruginosa* biofilms with comparable efficacy of dose-dependent concentrations. Tiopronin (20 mM) and tiopronin-NO (20 mM) inhibited 61.12 and 81.14% of the biofilm biomass formation in *E. coli*, respectively, compared to the untreated control (Figure S7a). Similarly, tiopronin showed 42.97% and tiopronin-NO showed 56.96% of biofilm growth inhibition against *P. aeruginosa* (Figure S7b). In both strains of bacteria, the drug showed sensitivity and inhibition of biofilm formation, demonstrated in the images of the CV-stained biomass (Figure S7c,d).

### Dissolution of Cystine Stones by Tiopronin-NO

Tiopronin is the most commonly used thiol-derived drug for the treatment of cystinuria.^7^*In vitro* studies were performed to show the effect (cystine binding to thiols) of tiopronin and tiopronin-NO on artificial urine, healthy patient urine, and a urine sample from a patient with cystinuria (Figure S8, details provided in Table S1). The study was conducted in different pH conditions to investigate tiopronin-NO activity and compare it to tiopronin’s previously documented pH-dependent activity in enhancing cystine solubility.^49,75,76^ To determine the kinetics of drug interaction (thiol exchange) in a healthy human urine sample, cystine dissolution was measured at different time points from 5 min to 48 h. The shortest time point of 5 min was investigated because it is most relevant to the actual amount of time that urine dwells within the renal pelvis, estimated to be about 5 min under high urine flow rate conditions.^77^ Previous studies have reported that cystine reaches equilibrium in urine at 37 °C within 48 h.^75,76^ Thus, in our studies, various time points within the therapeutic window were selected to evaluate the activity of tiopronin-NO in comparison with tiopronin.

**Figure 4:**
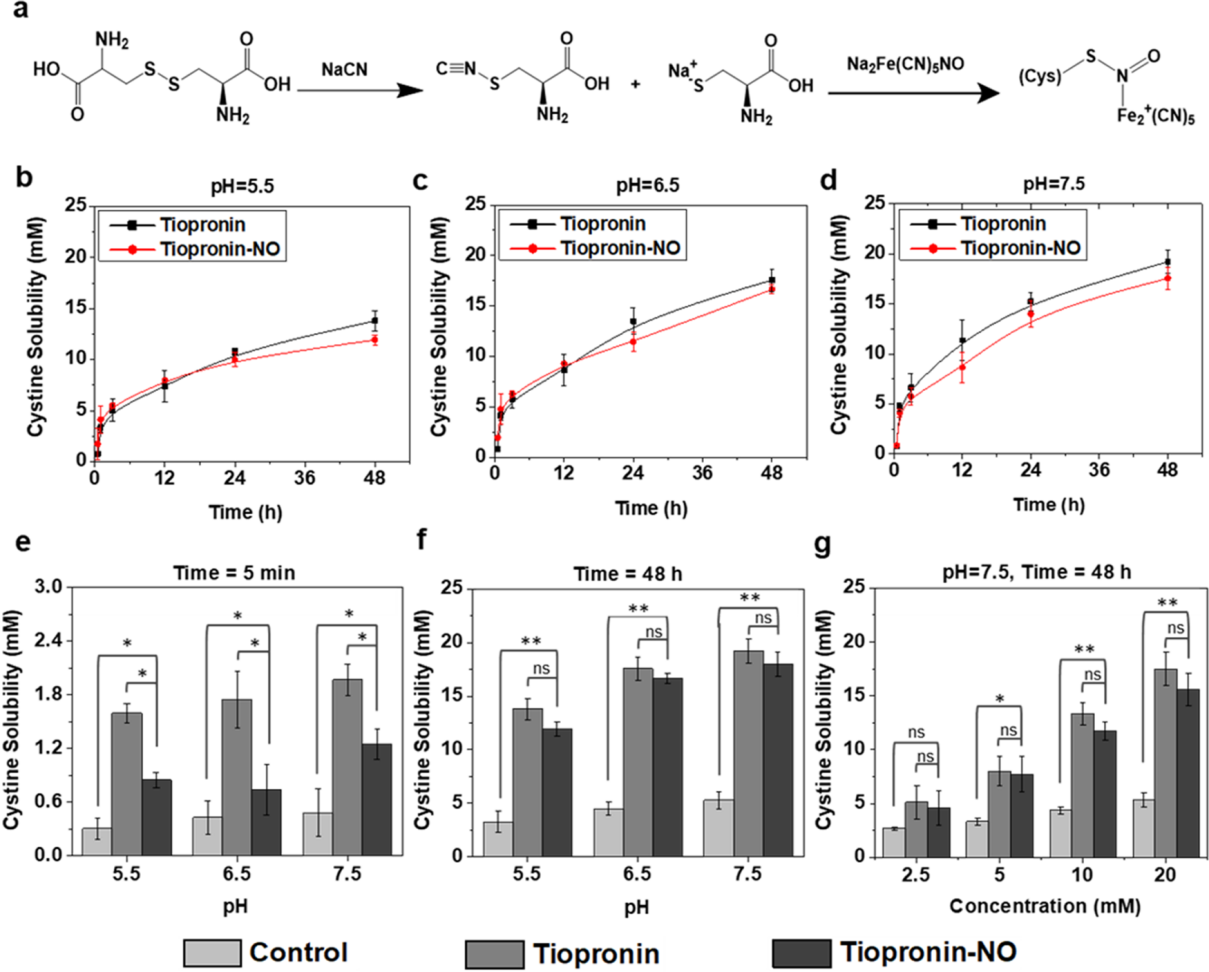
Cystine dissolution following tiopronin-NO treatment of artificial urine, urine from a healthy patient, and urine from a patient with cystinuria. (a) Cystine and sodium nitroprusside reaction scheme to quantify the cystine solubility in urine samples. (b–d) *In vitro* pH evaluation of cystine solubility in a urine sample from a healthy patient following treatment with tiopronin and tiopronin-NO. (e, f) Measurement of cystine solubility variation at different pH values and time points in artificial urine after incubation with cystine crystals and treatment with tiopronin and tiopronin-NO. Control represents the artificial urine sample without incubation of either drug. (g) *In vitro* measurement of cystine solubility in the urine of a patient with cystinuria following treatment with tiopronin and tiopronin-NO for 48 h and various concentrations of the drug. The results are shown as mean ± SD, and the asterisks represent a significant difference, *p < 0.05; **p < 0.01; ns, no significant difference.

The standard sodium nitroprusside assay (Figure 4a) quantifies cystine solubility in urine samples.^49,78^ During the study, it was found that as the pH increased from 5.5 to 7.5, cystine solubility in a healthy patient urine sample increased in the presence of both tiopronin and tiopronin-NO (Figure 4b–d). This indicates that thiol exchange occurs more quickly at pH 7.5 and reaches equilibrium at 48 h since alkali treatments break down cystine stones.^54^ After 5 min, both tiopronin and tiopronin-NO display enhanced cystine solubility in artificial urine compared to free cystine without treatment (Figure 4e). The slightly greater cystine solubility following treatment with tiopronin compared to tiopronin-NO can be explained by the delayed activity of tiopronin-NO due to the initial release of the NO molecule, which then exposes the sulfur group of the parent molecule for breaking of the cystine disulfide bond. Increasing the pH of the solution leads to greater NO release, more active thiol availability, and increased cystine solubility, as shown in the NO release data (Figure 1b). This dual function of tiopronin-NO means that the initial release of NO kills infection-causing pathogens, followed by the active thiol group breaking down the cystine stone with no loss of drug activity throughout 48 h (Figure 4f).

To mimic clinical applications, a cystinuria patient’s urine sample was treated with different concentrations of tiopronin and tiopronin-NO at pH 7.5 (Figure 4g). Under identical treatment conditions, the dose-dependent response of both the drugs enhanced cystine solubility compared to the control (healthy patient urine), which demonstrates that NO functionalization of tiopronin does not deter the characteristic properties of the drug.

To further evaluate this phenomenon, we analyzed the effect of both drugs on a urine sample through ionization mass spectroscopy and found the successful formation of a drug–cysteine complex. The masses *m*/*z* 375.2 and *m*/*z* 347.1 shown in Figure S9 indicate the formation of cystine complex with tiopronin (Figure S9a) and tiopronin-NO (Figure S9b) after interaction with cystine, as well as the formation of a dimer peak at *m*/*z* 726.9. Since no free compound peak is observed in the urine sample, this indicates the successful activity of the drugs to induce cystine solubilization after NO functionalization. Therefore, we conclude that tiopronin-NO shows dual therapeutic mechanisms in efficiently killing bacterial cells and reducing cystine stones, thus providing an alternative platform for the clinical treatment of cystinuria.

### *In Vitro* Biocompatibility Analysis

**Figure 5:**
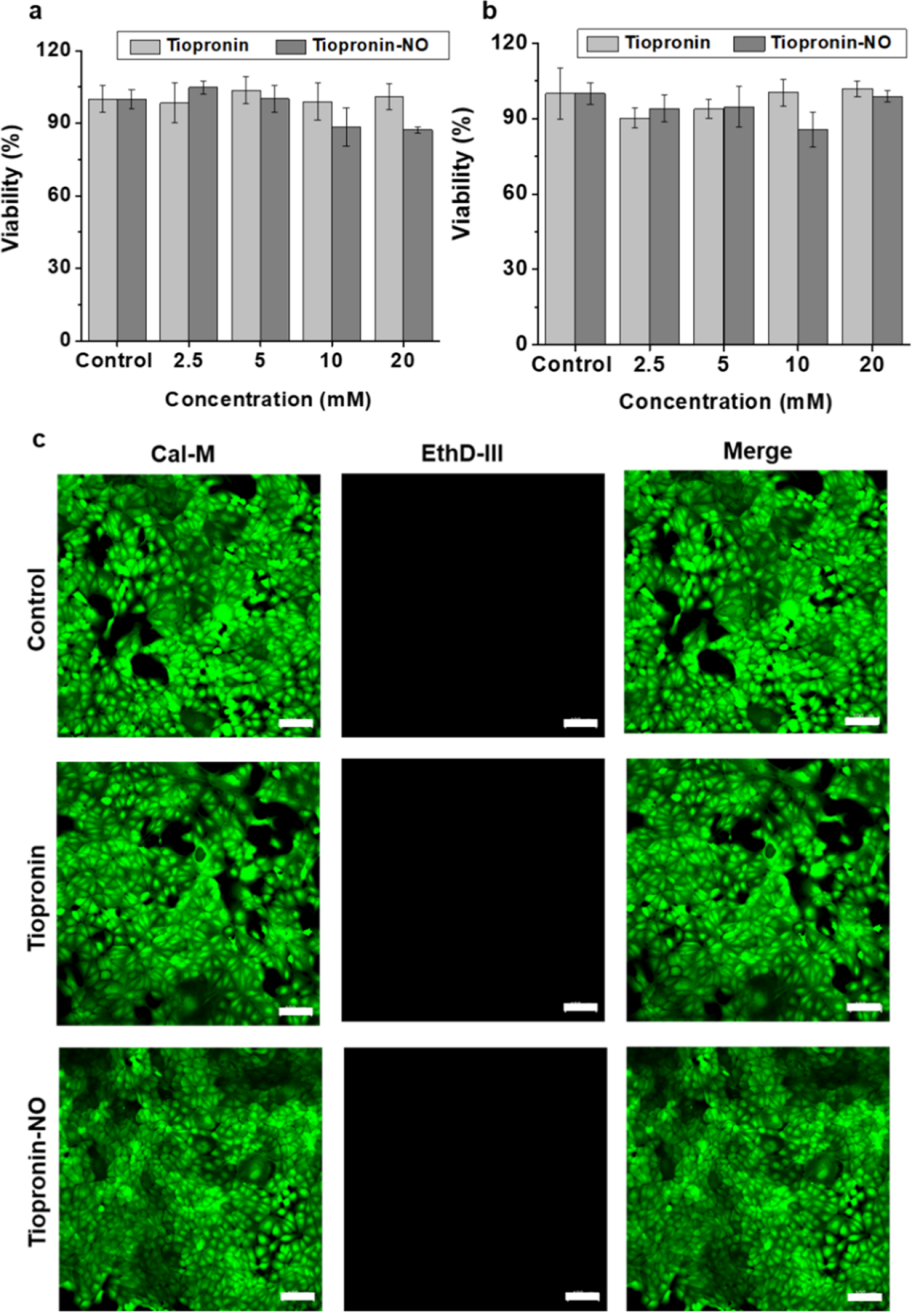
*In vitro* biocompatibility study. Effects of tiopronin and tiopronin-NO on canine kidney cells at (a) 24 h and (b) 72 h after treatment with various concentrations. (c) Live/dead cell imaging of the canine kidney cells treated with Tiopronin and Tiopronin-NO at a concentration of 20 mM for 24 h and imaged using a confocal laser scanning microscopy. The scale bar represents 100 μm. Data are expressed as the percentage of viability % ± SD of three independent experiments.

Nitric oxide is known for its diverse activities in biological system. In addition to acting as a potent antimicrobial, NO can also act as a signaling molecule to regulate body systems such as the vascular and nervous systems.^79^ Furthermore, NO affects cellular life and death decisions by turning apoptotic pathways on or shutting them off.^80^ This dual nature of NO depends upon the concentration and delivery method.^81,82^ Therefore, in our study, we further evaluated the toxicity of tiopronin and tiopronin-NO in two different cellular systems. For this, we selected a fibroblast cell (mouse) and a kidney cell (canine) to represent the bladder and kidneys, the most common organs infected during stone formation.^11^ The results of our study indicated that the cells were not adversely affected by either of the drugs at the desired concentrations (2.5, 5, 10, and 20 mM) for the 24 and 72 h viability studies (Figures 5a,b and S10a,b). To validate this finding, we employed confocal microscopy as a means of live cell imaging to assess the potential toxicity. For this, we used Calcein-AM as a marker for live cells and EthD-III as a probe for staining dead cells. We found that under both conditions, cells remained healthy, and no specific dead cells were observed when treated with tiopronin or tiopronin-NO as depicted in Figures 5c and S10c. Furthermore, the bright-field image analysis (Figures S11 and S12) confirmed that cell morphology was preserved, and no signs of toxicity were detected at the concentration utilized during the therapeutic study. Therefore, we conclude the newly synthesized tiopronin functionalized with NO is safe for use with mammalian cells.

## Conclusions

In summary, we developed a novel nitric oxide-releasing tiopronin conjugate prodrug (tiopronin-NO) from a well-known clinically used drug (tiopronin) for the treatment of cystinuria and bacterial infections surrounding the stone microenvironment. The dual therapeutic nature of the drug allows for the efficient killing of planktonic bacterial cells, and the dispersion and inhibition of the growth of biofilms, which are often found near the cystinuria stone microenvironment. In addition, after the release of NO, tiopronin-NO acts to reduce cystine stones through the disulfide bond formation with dispersed cysteine. At pH 7.5, tiopronin-NO greatly increases the rate of sulfide exchange with cystine to form soluble cysteine–drug complexes, which suggests that as the urine pH increases, the efficacy of the drug increases. The advanced dual-functional treatment was also found to be nontoxic to mouse fibroblast and canine kidney cells at the desired concentration used for antibacterial and cystine solubility studies. The utilized design strategy has enormous potential in inhibiting the growth of bacteria and biofilm formation, as well as reducing cystine stones combined into a single platform for the first time. Therefore, tiopronin-NO shows excellent promise for clinical use in managing and treating cystinuria. The findings establish a structural framework for utilizing *in vitro* data to predict the *in vivo* efficacy of drugs for treating and managing cystinuria. Further clinical investigations are required to assess the therapeutic and safety aspects of the drug for human use.

## Supporting Information Available

Schematic diagram of nitrosation reaction; ^1^H NMR spectra of tiopronin and tiopronin-NO; mass spectrometry of tiopronin-NO; UV–vis and FTIR spectra of tiopronin and tiopronin-NO; NO localization in bacteria treated with tiopronin and tiopronin-NO; 3D images of biofilms treated with tiopronin and tiopronin-NO; SEM images of *E. coli* and *P. aeruginosa* biofilms treated with tiopronin and tiopronin-NO; crystal violet assay of biofilms treated with tiopronin and tiopronin-NO; images of urine samples used for cystine studies; table of urine sample properties and contents; mass analysis of urine samples treated with tiopronin and tiopronin-NO; cytocompatibility tests using mouse fibroblast cells; bright-field microscopy images of canine kidney cells following treatment with tiopronin and tiopronin-NO; and bright-field microscopy images of mouse fibroblast cells following treatment with tiopronin and tiopronin-NO (PDF)

## References

[ref1] Scales C. D. Jr.; Smith A. C.; Hanley J. M.; Saigal C. S. Prevalence of kidney stones in the United States. Eur. Urol. 2012, 62 (1), 160–165. 10.1016/j.eururo.2012.03.052. 22498635 PMC3362665

[ref2] Chillarón J.; Font-Llitjós M.; Fort J.; Zorzano A.; Goldfarb D. S.; Nunes V.; Palacín M. Pathophysiology and treatment of cystinuria. Nat. Rev. Nephrol. 2010, 6 (7), 424–434. 10.1038/nrneph.2010.69. 20517292

[ref3] Thomas K.; Wong K.; Withington J.; Bultitude M.; Doherty A. Cystinuria—a urologist’s perspective. Nat. Rev. Urol. 2014, 11 (5), 270–277. 10.1038/nrurol.2014.51. 24662732

[ref4] Rimer J. D.; An Z.; Zhu Z.; Lee M. H.; Goldfarb D. S.; Wesson J. A.; Ward M. D. Crystal growth inhibitors for the prevention of L-cystine kidney stones through molecular design. Science 2010, 330 (6002), 337–341. 10.1126/science.1191968. 20947757 PMC5166609

[ref5] Shtukenberg A. G.; Hu L.; Sahota A.; Kahr B.; Ward M. D. Disrupting crystal growth through molecular recognition: designer therapies for kidney stone prevention. Acc. Chem. Res. 2022, 55 (4), 516–525. 10.1021/acs.accounts.1c00631. 35088591 PMC10355143

[ref6] Zee T.; Bose N.; Zee J.; Beck J. N.; Yang S.; Parihar J.; Yang M.; Damodar S.; Hall D.; O’Leary M. N.; et al. α-Lipoic acid treatment prevents cystine urolithiasis in a mouse model of cystinuria. Nat. Med. 2017, 23 (3), 288–290. 10.1038/nm.4280. 28165480 PMC5656064

[ref7] Moussa M.; Papatsoris A. G.; Abou Chakra M.; Moussa Y. Update on cystine stones: current and future concepts in treatment. Intractable Rare Dis. Res. 2020, 9 (2), 71–78. 10.5582/irdr.2020.03006. 32494553 PMC7263987

[ref8] Kumar A.; Ma H.; Zhang X.; Huang K.; Jin S.; Liu J.; Wei T.; Cao W.; Zou G.; Liang X.-J. Gold nanoparticles functionalized with therapeutic and targeted peptides for cancer treatment. Biomaterials 2012, 33 (4), 1180–1189. 10.1016/j.biomaterials.2011.10.058. 22056754

[ref9] Espinosa-Ortiz E. J.; Eisner B. H.; Lange D.; Gerlach R. Current insights into the mechanisms and management of infection stones. Nat. Rev. Urol. 2019, 16 (1), 35–53. 10.1038/s41585-018-0120-z. 30470787

[ref10] Miller A. W.; Penniston K. L.; Fitzpatrick K.; Agudelo J.; Tasian G.; Lange D. Mechanisms of the intestinal and urinary microbiome in kidney stone disease. Nat. Rev. Urol. 2022, 19 (12), 695–707. 10.1038/s41585-022-00647-5. 36127409 PMC11234243

[ref11] Flannigan R.; Choy W. H.; Chew B.; Lange D. Renal Struvite Stones—Pathogenesis, Microbiology, and Management Strategies. Nat. Rev. Urol. 2014, 11 (6), 333–341. 10.1038/nrurol.2014.99. 24818849

[ref12] Alford A.; Furrow E.; Borofsky M.; Lulich J. Animal models of naturally occurring stone disease. Nat. Rev. Urol. 2020, 17 (12), 691–705. 10.1038/s41585-020-00387-4. 33159170 PMC9254658

[ref13] Schwaderer A. L.; Wolfe A. J. The association between bacteria and urinary stones. Ann. Transl. Med. 2017, 5 (2), 32 10.21037/atm.2016.11.73. 28217697 PMC5300853

[ref14] Armbruster C. E.; Mobley H. L.; Pearson M. M., Pathogenesis of *Proteus mirabilis* infection. EcoSal Plus 2018, 8 ( (1), ). 10.1128/ecosalplus.ESP-0009-2017. PMC5880328 29424333

[ref15] Thomas B.; Tolley D. Concurrent urinary tract infection and stone disease: pathogenesis, diagnosis and management. Nat. Clin. Pract. Urol. 2008, 5 (12), 668–675. 10.1038/ncpuro1254. 19050709

[ref16] Blair J. M. A.; Webber M. A.; Baylay A. J.; Ogbolu D. O.; Piddock L. J. V. Molecular Mechanisms of Antibiotic Resistance. Nat. Rev. Microbiol. 2015, 13 (1), 42–51. 10.1038/nrmicro3380. 25435309

[ref17] Mancuso G.; Midiri A.; Gerace E.; Biondo C. Bacterial Antibiotic Resistance: The Most Critical Pathogens. Pathogens 2021, 10 (10), 1310 10.3390/pathogens10101310. 34684258 PMC8541462

[ref18] Gebreyohannes G.; Nyerere A.; Bii C.; Sbhatu D. B. Challenges of Intervention, Treatment, and Antibiotic Resistance of Biofilm-Forming Microorganisms. Heliyon 2019, 5 (8), e02192 10.1016/j.heliyon.2019.e02192. 31463386 PMC6709409

[ref19] Høiby N.; Bjarnsholt T.; Givskov M.; Molin S.; Ciofu O. Antibiotic Resistance of Bacterial Biofilms. Int. J. Antimicrob. Agents 2010, 35 (4), 322–332. 10.1016/j.ijantimicag.2009.12.011. 20149602

[ref20] Carpenter A. W.; Schoenfisch M. H. Nitric oxide release: Part II. Therapeutic applications. Chem. Soc. Rev. 2012, 41 (10), 3742–3752. 10.1039/c2cs15273h. 22362384 PMC3341526

[ref21] Mishra B. B.; Lovewell R. R.; Olive A. J.; Zhang G.; Wang W.; Eugenin E.; Smith C. M.; Phuah J. Y.; Long J. E.; Dubuke M. L.; et al. Nitric oxide prevents a pathogen-permissive granulocytic inflammation during tuberculosis. Nat. Microbiol. 2017, 2 (7), 1–11. 10.1038/nmicrobiol.2017.72. PMC5461879 28504669

[ref22] Chen Z.; Zheng S.; Shen Z.; Cheng J.; Xiao S.; Zhang G.; Liu S.; Hu J. Oxygen-Tolerant Photoredox Catalysis Triggers Nitric Oxide Release for Antibacterial Applications. Angew. Chem., Int. Ed. 2022, 61 (30), e202204526 10.1002/anie.202204526. 35579256

[ref23] Bao X.; Zheng S.; Zhang L.; Shen A.; Zhang G.; Liu S.; Hu J. Nitric-Oxide-Releasing aza-BODIPY: A New Near-Infrared J-Aggregate with Multiple Antibacterial Modalities. Angew. Chem., Int. Ed. 2022, 61 (32), e202207250 10.1002/anie.202207250. 35657486

[ref24] Gao L.; Cheng J.; Shen Z.; Zhang G.; Liu S.; Hu J. Orchestrating Nitric Oxide and Carbon Monoxide Signaling Molecules for Synergistic Treatment of MRSA Infections. Angew. Chem., Int. Ed. 2022, 61 (3), e202112782. 10.1002/anie.202112782 34694047

[ref25] Änggård E. Nitric oxide: mediator, murderer, and medicine. Lancet 1994, 343 (8907), 1199–1206. 10.1016/S0140-6736(94)92405-8. 7909873

[ref26] Gao S.; Zhang W.; Wang R.; Hopkins S. P.; Spagnoli J. C.; Racin M.; Bai L.; Li L.; Jiang W.; Yang X.; et al. Nanoparticles encapsulating nitrosylated maytansine to enhance radiation therapy. ACS Nano 2020, 14 (2), 1468–1481. 10.1021/acsnano.9b05976. 31939662

[ref27] Kumar A.; Mondal A.; Douglass M. E.; Francis D. J.; Garren M. R.; Estes Bright L. M.; Ghalei S.; Xie J.; Brisbois E. J.; Handa H. Nanoarchitectonics of nitric oxide releasing supramolecular structures for enhanced antibacterial efficacy under visible light irradiation. J. Colloid Interface Sci. 2023, 640, 144–161. 10.1016/j.jcis.2023.02.083. 36842420 PMC10081829

[ref28] Barraud N.; Schleheck D.; Klebensberger J.; Webb J. S.; Hassett D. J.; Rice S. A.; Kjelleberg S. Nitric Oxide Signaling in Pseudomonas aeruginosa Biofilms Mediates Phosphodiesterase Activity, Decreased Cyclic di-GMP Levels, and Enhanced Dispersal. J. Bacteriol. 2009, 191 (23), 7333–7342. 10.1128/JB.00975-09. 19801410 PMC2786556

[ref29] Barraud N.; Hassett D. J.; Hwang S.-H.; Rice S. A.; Kjelleberg S.; Webb J. S. Involvement of Nitric Oxide in Biofilm Dispersal of Pseudomonas aeruginosa. J. Bacteriol. 2006, 188 (21), 7344–7353. 10.1128/JB.00779-06. 17050922 PMC1636254

[ref30] Barraud N.; J Kelso M.; A Rice S.; Kjelleberg S. Nitric Oxide: a Key Mediator of Biofilm Dispersal with Applications in Infectious Diseases. Curr. Pharm. Des. 2014, 21 (1), 31–42. 10.2174/1381612820666140905112822. 25189865

[ref31] Mondal A.; Douglass M.; Hopkins S. P.; Singha P.; Tran M.; Handa H.; Brisbois E. J. Multifunctional S-Nitroso-N-acetylpenicillamine-Incorporated Medical-Grade Polymer with Selenium Interface for Biomedical Applications. ACS Appl. Mater. Interfaces 2019, 11 (38), 34652–34662. 10.1021/acsami.9b10610. 31483604 PMC8007129

[ref32] Estes Bright L. M.; Garren M. R.; Ashcraft M.; Kumar A.; Husain H.; Brisbois E. J.; Handa H. Dual Action Nitric Oxide and Fluoride Ion-Releasing Hydrogels for Combating Dental Caries. ACS Appl. Mater. Interfaces 2022, 14 (19), 21916–21930. 10.1021/acsami.2c02301. 35507415

[ref33] Chug M. K.; Brisbois E. J. Recent Developments in Multifunctional Antimicrobial Surfaces and Applications toward Advanced Nitric Oxide-Based Biomaterials. ACS Mater. Au 2022, 2 (5), 525–551. 10.1021/acsmaterialsau.2c00040. 36124001 PMC9479141

[ref34] Rouillard K. R.; Novak O. P.; Pistiolis A. M.; Yang L.; Ahonen M. J.; McDonald R. A.; Schoenfisch M. H. Exogenous nitric oxide improves antibiotic susceptibility in resistant bacteria. ACS Infect. Dis. 2021, 7 (1), 23–33. 10.1021/acsinfecdis.0c00337. 33291868

[ref35] Brisbois E. J.; Davis R. P.; Jones A. M.; Major T. C.; Bartlett R. H.; Meyerhoff M. E.; Handa H. Reduction in thrombosis and bacterial adhesion with 7 day implantation of S-nitroso-N-acetylpenicillamine (SNAP)-doped Elast-eon E2As catheters in sheep. J. Mater. Chem. B 2015, 3 (8), 1639–1645. 10.1039/C4TB01839G. 25685358 PMC4326019

[ref36] Brisbois E. J.; Handa H.; Major T. C.; Bartlett R. H.; Meyerhoff M. E. Long-term nitric oxide release and elevated temperature stability with S-nitroso-N-acetylpenicillamine (SNAP)-doped Elast-eon E2As polymer. Biomaterials 2013, 34 (28), 6957–6966. 10.1016/j.biomaterials.2013.05.063. 23777908 PMC3729938

[ref37] de Oliveira M. G.; Shishido S. M.; Seabra A. B.; Morgon N. H. Thermal stability of primary S-nitrosothiols: roles of autocatalysis and structural effects on the rate of nitric oxide release. J. Phys. Chem. A 2002, 106 (38), 8963–8970. 10.1021/jp025756u.

[ref38] Hogg N. Biological chemistry and clinical potential of S-nitrosothiols. Free Radical Biol. Med. 2000, 28 (10), 1478–1486. 10.1016/S0891-5849(00)00248-3. 10927172

[ref39] Williams D. L. H. The chemistry of S-nitrosothiols. Acc. Chem. Res. 1999, 32 (10), 869–876. 10.1021/ar9800439.

[ref40] Bartberger M. D.; Mannion J. D.; Powell S. C.; Stamler J. S.; Houk K.; Toone E. J. S– N dissociation energies of S-nitrosothiols: on the origins of nitrosothiol decomposition rates. J. Am. Chem. Soc. 2001, 123 (36), 8868–8869. 10.1021/ja0109390. 11535101

[ref41] Nagy P. Kinetics and mechanisms of thiol–disulfide exchange covering direct substitution and thiol oxidation-mediated pathways. Antioxid. Redox Signaling 2013, 18 (13), 1623–1641. 10.1089/ars.2012.4973. PMC3613173 23075118

[ref42] Hopkins S. P.; Frost M. C. Synthesis and Characterization of Controlled Nitric Oxide Release from S-Nitroso-N-Acetyl-d-Penicillamine Covalently Linked to Polyvinyl Chloride (SNAP-PVC). Bioengineering 2018, 5 (3), 72 10.3390/bioengineering5030072. 30189614 PMC6165297

[ref43] Pore R. S. Antibiotic susceptibility testing by flow cytometry. J. Antimicrob. Chemother. 1994, 34 (5), 613–627. 10.1093/jac/34.5.613. 7706157

[ref44] Robertson J.; McGoverin C.; Vanholsbeeck F.; Swift S. Optimisation of the Protocol for the LIVE/DEAD BacLightTM Bacterial Viability Kit for Rapid Determination of Bacterial Load. Front. Microbiol. 2019, 10, 801 10.3389/fmicb.2019.00801. 31031741 PMC6474257

[ref45] Gao S.; Zhang W.; Wang R.; Hopkins S. P.; Spagnoli J. C.; Racin M.; Bai L.; Li L.; Jiang W.; Yang X.; Lee C.; Nagata K.; Howerth E. W.; Handa H.; Xie J.; Ma Q.; Kumar A. Nanoparticles Encapsulating Nitrosylated Maytansine To Enhance Radiation Therapy. ACS Nano 2020, 14 (2), 1468–1481. 10.1021/acsnano.9b05976. 31939662

[ref46] Jurcisek J. A.; Dickson A. C.; Bruggeman M. E.; Bakaletz L. O. In vitro Biofilm Formation in an 8-well Chamber Slide. J. Visualized Exp. 2011, (47), 2481 10.3791/2481-v. PMC3182645 21304464

[ref47] Coffey B. M.; Anderson G. G. Biofilm formation in the 96-well microtiter plate. In Methods in Molecular Biology; Springer: New York, 2014; Vol. 1149, pp 631–641. 10.1007/978-1-4939-0473-0_48. 24818938

[ref48] Li E.; Mira de Orduña R. A rapid method for the determination of microbial biomass by dry weight using a moisture analyser with an infrared heating source and an analytical balance. Lett. Appl. Microbiol. 2010, 50 (3), 283–288. 10.1111/j.1472-765X.2009.02789.x. 20070868

[ref49] Asplin D. M.; Asplin J. R. The Interaction of Thiol Drugs and Urine pH in the Treatment of Cystinuria. J. Urol. 2013, 189 (6), 2147–2151. 10.1016/j.juro.2012.12.031. 23261477

[ref50] Nakagawa Y.; Coe F. L. A modified cyanide-nitroprusside method for quantifying urinary cystine concentration that corrects for creatinine interference. Clin. Chim. Acta 1999, 289 (1–2), 57–68. 10.1016/S0009-8981(99)00159-X. 10556653

[ref51] Goldfarb D. S.; Coe F. L.; Asplin J. R. Urinary cystine excretion and capacity in patients with cystinuria. Kidney Int. 2006, 69 (6), 1041–1047. 10.1038/sj.ki.5000104. 16501494

[ref52] Azeez S.; Chaudhary P.; Sureshbabu P.; Sabiah S.; Kandasamy J. tert-Butyl nitrite mediated nitrogen transfer reactions: synthesis of benzotriazoles and azides at room temperature. Org. Biomol. Chem. 2018, 16 (37), 8280–8285. 10.1039/C8OB01950A. 30209482

[ref53] Cai X.; Chen H.-H.; Wang C.-L.; Chen S.-T.; Lai S.-F.; Chien C.-C.; Chen Y.-Y.; Kempson I. M.; Hwu Y.; Yang C. S.; Margaritondo G. Imaging the cellular uptake of tiopronin-modified gold nanoparticles. Anal. Bioanal. Chem. 2011, 401 (3), 809–816. 10.1007/s00216-011-4986-3. 21537916

[ref54] Servais A.; Thomas K.; Dello Strologo L.; Sayer J. A.; Bekri S.; Bertholet-Thomas A.; Bultitude M.; Capolongo G.; Cerkauskiene R.; Daudon M.; Doizi S.; Gillion V.; Gràcia-Garcia S.; Halbritter J.; Heidet L.; van den Heijkant M.; Lemoine S.; Knebelmann B.; Emma F.; Levtchenko E. Cystinuria: clinical practice recommendation. Kidney Int. 2021, 99 (1), 48–58. 10.1016/j.kint.2020.06.035. 32918941

[ref55] Moussa M.; Papatsoris A. G.; Abou Chakra M.; Moussa Y. Update on cystine stones: current and future concepts in treatment. Intractable Rare Dis Res. 2020, 9 (2), 71–78. 10.5582/irdr.2020.03006. 32494553 PMC7263987

[ref56] D’Ambrosio V.; Capolongo G.; Goldfarb D.; Gambaro G.; Ferraro P. M. Cystinuria: An Update on Pathophysiology, Genetics, and Clinical Management. Pediatr. Nephrol. 2022, 37 (8), 1705–1711. 10.1007/s00467-021-05342-y. 34812923

[ref57] Li W.; Wang D.; Lao K. U.; Wang X. Buffer concentration dramatically affects the stability of S-nitrosothiols in aqueous solutions. Nitric Oxide 2022, 118, 59–65. 10.1016/j.niox.2021.11.002. 34848361

[ref58] Zhang C.; Biggs T. D.; Devarie-Baez N. O.; Shuang S.; Dong C.; Xian M. S-Nitrosothiols: chemistry and reactions. Chem. Commun. 2017, 53 (82), 11266–11277. 10.1039/C7CC06574D. 28944382

[ref59] Melvin A. C.; Jones W. M.; Lutzke A.; Allison C. L.; Reynolds M. M. S-Nitrosoglutathione Exhibits Greater Stability Than S-nitroso-N-acetylpenicillamine Under Common Laboratory Conditions: A Comparative Stability Study. Nitric Oxide 2019, 92, 18–25. 10.1016/j.niox.2019.08.002. 31398487

[ref60] Williams D. L. H. The chemistry of S-nitrosothiols. Acc. Chem. Res. 1999, 32 (10), 869 10.1021/ar9800439.

[ref61] Xie J.; Huang J.-S.; Huang X.-J.; Peng J.-M.; Yu Z.; Yuan Y.-Q.; Xiao K.-F.; Guo J.-N. Profiling the urinary microbiome in men with calcium-based kidney stones. BMC Microbiol. 2020, 20 (1), 41 10.1186/s12866-020-01734-6. 32111156 PMC7049185

[ref62] Chetlapalli S. S. Tiopronin Oral Composition. 2019.

[ref63] Munday R. Toxicity of thiols and disulphides: Involvement of free-radical species. Free Radical Biol. Med. 1989, 7 (6), 659–673. 10.1016/0891-5849(89)90147-0. 2695409

[ref64] De Groote M. A.; Fang F. C. NO Inhibitions: Antimicrobial Properties of Nitric Oxide. Clin. Infect. Dis. 1995, 21 (Supplement_2), S162–S165. 10.1093/clinids/21.Supplement_2.S162. 8845445

[ref65] Fang F. C. Perspectives Series: Host/Pathogen Interactions. Mechanisms of Nitric Oxide-Related Antimicrobial Activity. J. Clin. Invest. 1997, 99 (12), 2818–2825. 10.1172/JCI119473. 9185502 PMC508130

[ref66] Fang F. C. Antimicrobial Reactive Oxygen and Nitrogen Species: Concepts and Controversies. Nat. Rev. Microbiol. 2004, 2 (10), 820–832. 10.1038/nrmicro1004. 15378046

[ref67] Caron G. N.-V.; Stephens; Badley Assessment of bacterial viability status by flow cytometry and single cell sorting. J. Appl. Microbiol. 1998, 84 (6), 988–998. 10.1046/j.1365-2672.1998.00436.x. 9717283

[ref68] Lewis A.; Matzdorf S.; Rice K. Fluorescent Detection of Intracellular Nitric Oxide in *Staphylococcus aureus*. Bio-Protoc. 2016, 6 (14), e1878 10.21769/BioProtoc.1878. 28491909 PMC5421646

[ref69] Choong S.; Whitfield H. Biofilms and their role in infections in urology. BJU Int. 2001, 86 (8), 935–941. 10.1046/j.1464-410x.2000.00949.x. 11069430

[ref70] Singh S.; Singh S. K.; Chowdhury I.; Singh R. Understanding the Mechanism of Bacterial Biofilms Resistance to Antimicrobial Agents. Open Microbiol. J. 2017, 11, 53–62. 10.2174/1874285801711010053. 28553416 PMC5427689

[ref71] Merritt J. H.; Kadouri D. E.; O’Toole G. A. Growing and Analyzing Static Biofilms. Current Protocols in Microbiology 2011, 22 (1), 1B.1.1–1B.1.18. 10.1002/9780471729259.mc01b01s22. PMC4568995 18770545

[ref72] Li Y.; Heine S.; Entian M.; Sauer K.; Frankenberg-Dinkel N. NO-Induced Biofilm Dispersion in Pseudomonas aeruginosa Is Mediated by an MHYT Domain-Coupled Phosphodiesterase. J. Bacteriol. 2013, 195 (16), 3531–3542. 10.1128/jb.01156-12. 23729646 PMC3754559

[ref73] Thibeaux R.; Kainiu M.; Goarant C. Biofilm Formation and Quantification Using the 96-Microtiter Plate. In Leptospira spp.: Methods and Protocols; Koizumi N.; Picardeau M., Eds.; Springer: New York, NY, 2020; pp 207–214. 10.1007/978-1-0716-0459-5_19 32632872

[ref74] Silva S.; Henriques M.; Martins A.; Oliveira R.; Williams D.; Azeredo J. Biofilms of non-Candida albicans Candidaspecies: quantification, structure and matrix composition. Med. Mycol. 2009, 47 (7), 681–689. 10.3109/13693780802549594. 19888800

[ref75] Nakagawa Y.; Asplin J. R.; Goldfarb D. S.; Parks J. H.; Coe F. L. Clinical use of cystine supersaturation measurements. J. Urol. 2000, 164 (5), 1481–1485. 10.1016/S0022-5347(05)67011-5. 11025687

[ref76] Pak C. Y.; Fuller C. J. Assessment of Cystine Solubility in Urine and of Heterogeneous Nucleation. Journal of Urology 1983, 129 (5), 1066–1070. 10.1016/S0022-5347(17)52543-4. 6854756

[ref77] Finlayson B.; Reid F. The expectation of free and fixed particles in urinary stone disease. Invest Urol. 1978, 15 (6), 442–448. 649291

[ref78] Brady C. T.; Giesen C. D.; Voskoboev N.; Chirackal R. S.; Gavrilov D. K.; Flanagan R. M.; Lieske J. C. Cyanide-Nitroprusside Colorimetric Assay: A Rapid Colorimetric Screen for Urinary Cystine. J. Appl. Lab. Med. 2017, 2 (1), 55–64. 10.1373/jalm.2016.022582. 33636959

[ref79] Levine A. B.; Punihaole D.; Levine T. B. Characterization of the Role of Nitric Oxide and Its Clinical Applications. Cardiology 2012, 122 (1), 55–68. 10.1159/000338150. 22722323

[ref80] Brüne B. Nitric Oxide: NO Apoptosis or Turning it ON?. Cell Death Differ. 2003, 10 (8), 864–869. 10.1038/sj.cdd.4401261. 12867993

[ref81] Kumar A.; Radzievski R.; Xie J. Free Radical: Nitric Oxide in Cancer Therapy. Int. J. Anal. Med. Chem. 2018, 1, 1–4.

[ref82] Bryan N. S. Nitric oxide enhancement strategies. Future Sci. OA 2015, 1 (1), FSO48 10.4155/fso.15.48. 28031863 PMC5137939

