# Supplementary material for: Chemical Modification of Tiopronin for Dual Management of Cystinuria and Associated Bacterial Infections
Source: ACS Appl Mater Interfaces. 2023 Sep 6;15(37):43332–44. doi: 10.1021/acsami.3c07160 (PMC10520916; doi:10.1021/acsami.3c07160)
Supplement: Supplementary file 1 — am3c07160_si_001.pdf [file am3c07160_si_001.pdf]

Supporting Information

Chemical modification of tiopronin for dual management of cystinuria and associated bacterial infections

*Anil Kumar,[1¶] Lori M. Estes Bright,[1¶] [1]Mark Richard Stephen Garren[1], James Manuel[1], Arpita Shome[1], and Hitesh Handa[1,2 *]*

[1]School of Chemical Materials and Biomedical Engineering, University of Georgia, Athens, Georgia 30602, United States

[2]Pharmaceutical and Biomedical Sciences Department, College of Pharmacy, University of Georgia, Athens, GA 30602, United States

¶ ***A. K. and L.E.B.*** contributed equally to this work.

Address Correspondence to:
Dr. Hitesh Handa
College of Engineering, University of Georgia,
iSTEM 2, 302 E Campus Rd, Room # 2214
Athens, GA 30602
Email: hhanda@uga.edu
Office Phone: 706-542-8109

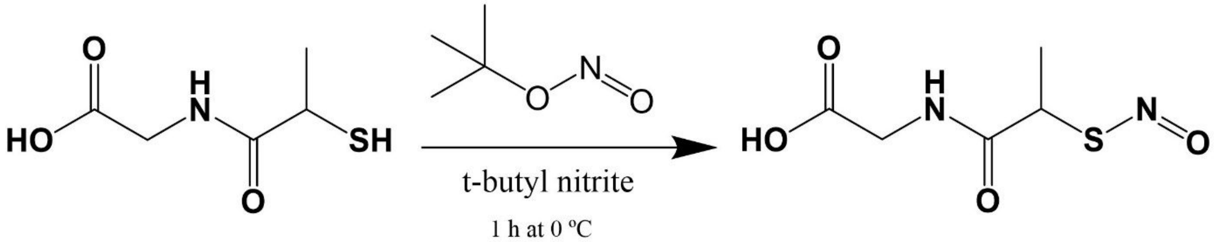

Scheme S1. Schematic diagram showing the reaction scheme for the synthesis of S-nitroso-2-mercaptopropionyl glycine.

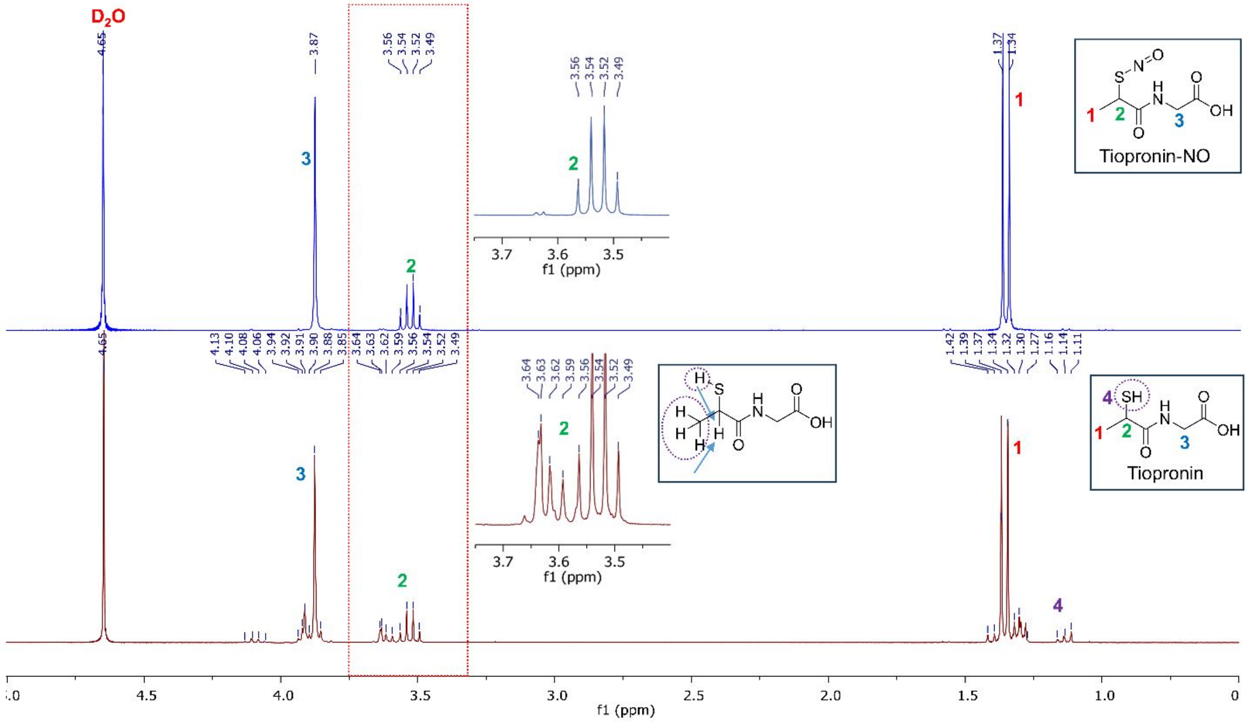

Figure S1. ^{1}H NMR spectra of tiopronin-NO prodrug (top) and tiopronin (bottom) in deuterated NMR grade water (D2O). Proton at 2 has 3 & 1 neighboring protons. That influences the peak of 2, leading to complex coupling and a split of (3+1) x (1+1) = 8. After nitrosation, the peak number reduced to 4 which shows the absence of a proton at sulfur, which is replaced by the NO group. Nitrosation is further evidenced by loss of peak at 4 from reduced thiol.

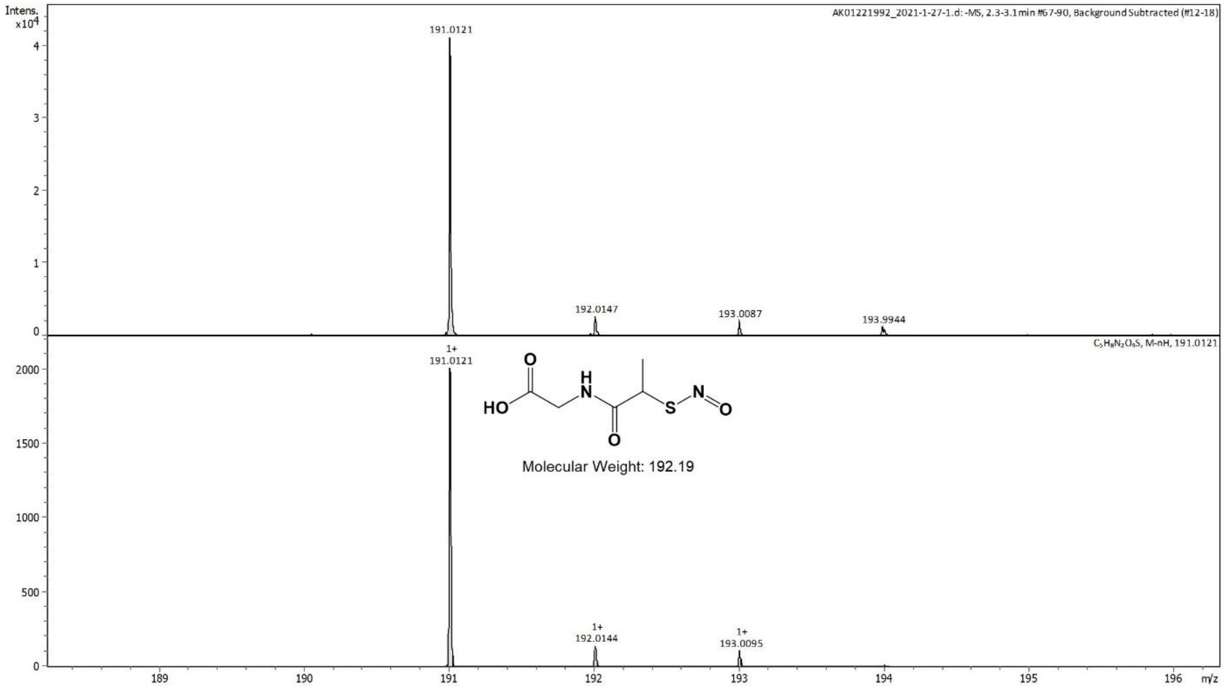

Figure S2. High-resolution mass spectrometry (HRMS) analysis of S-nitroso-2-mercaptopropionyl glycine (tiopronin-NO)

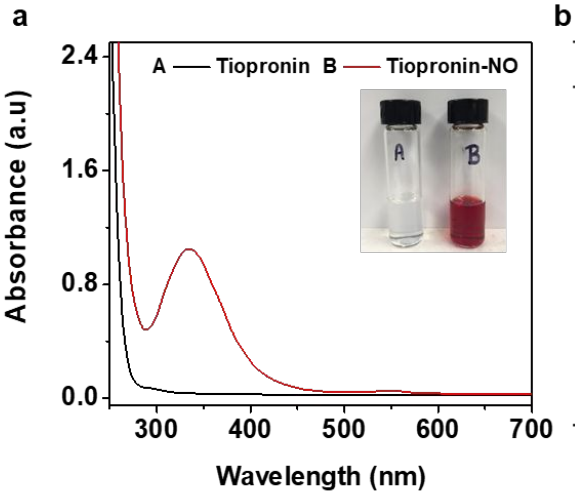

b

#	TIOP	TIOP-NO	Assignment
i	3317 (s)	3370 (br)	Amide A
ii	2939 (w)	2935 (w)	Amide B
iii	2566 (w)	- -	S-H Stretching
iv	1752 (s)	1735 (br)	C=O Stretching
v	1622 (s)	1650 (br)	Amide I
vi	1557 (m)	1537 (br)	Amide II & N=O Stretching
vii	- -	657 (br)	-S-S Stretching

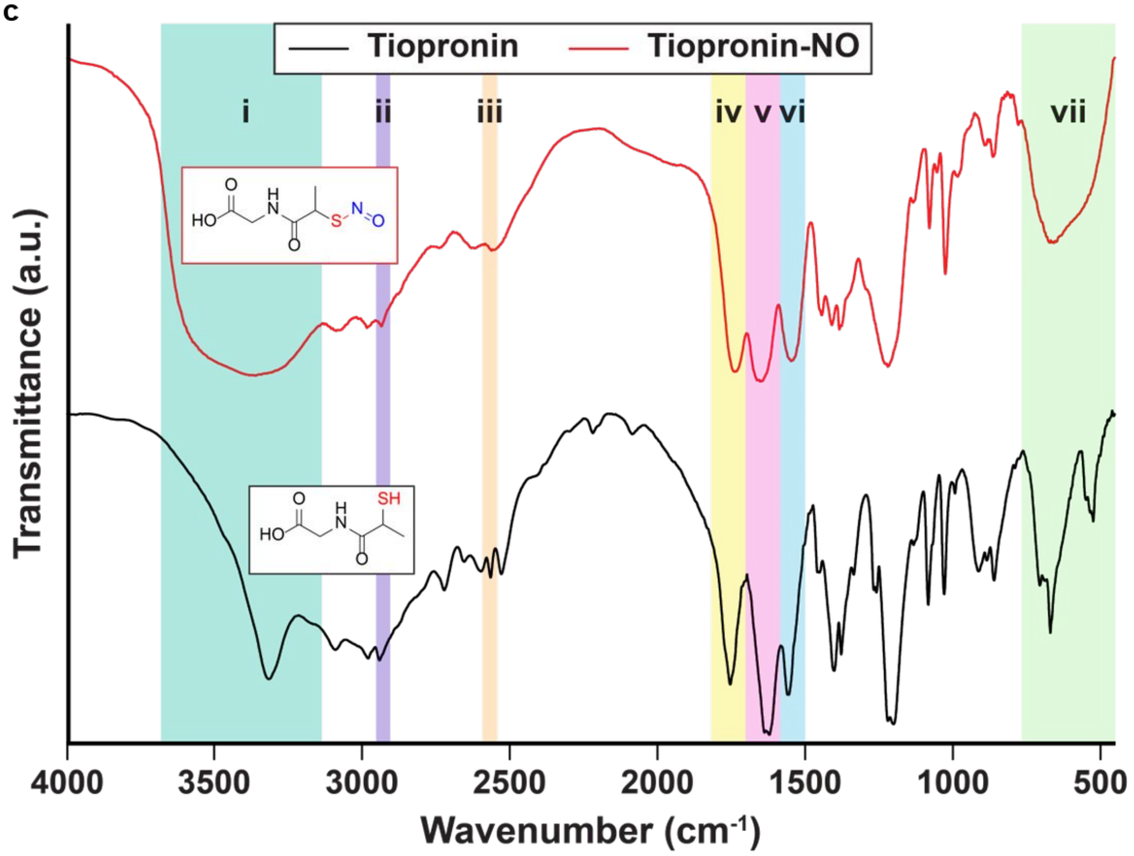

Figure S3. (a) UV spectrum of tiopronin and tiopronin-NO in aqueous solution at 37 °C. The inset images A and B indicate the solution of tiopronin and tiopronin-NO in water. The same solution was used for spectroscopy analysis. (b) Highlighted Fourier transform infrared spectroscopy (FTIR) band assignments and corresponding (c) FTIR spectra of tiopronin and tiopronin-NO.

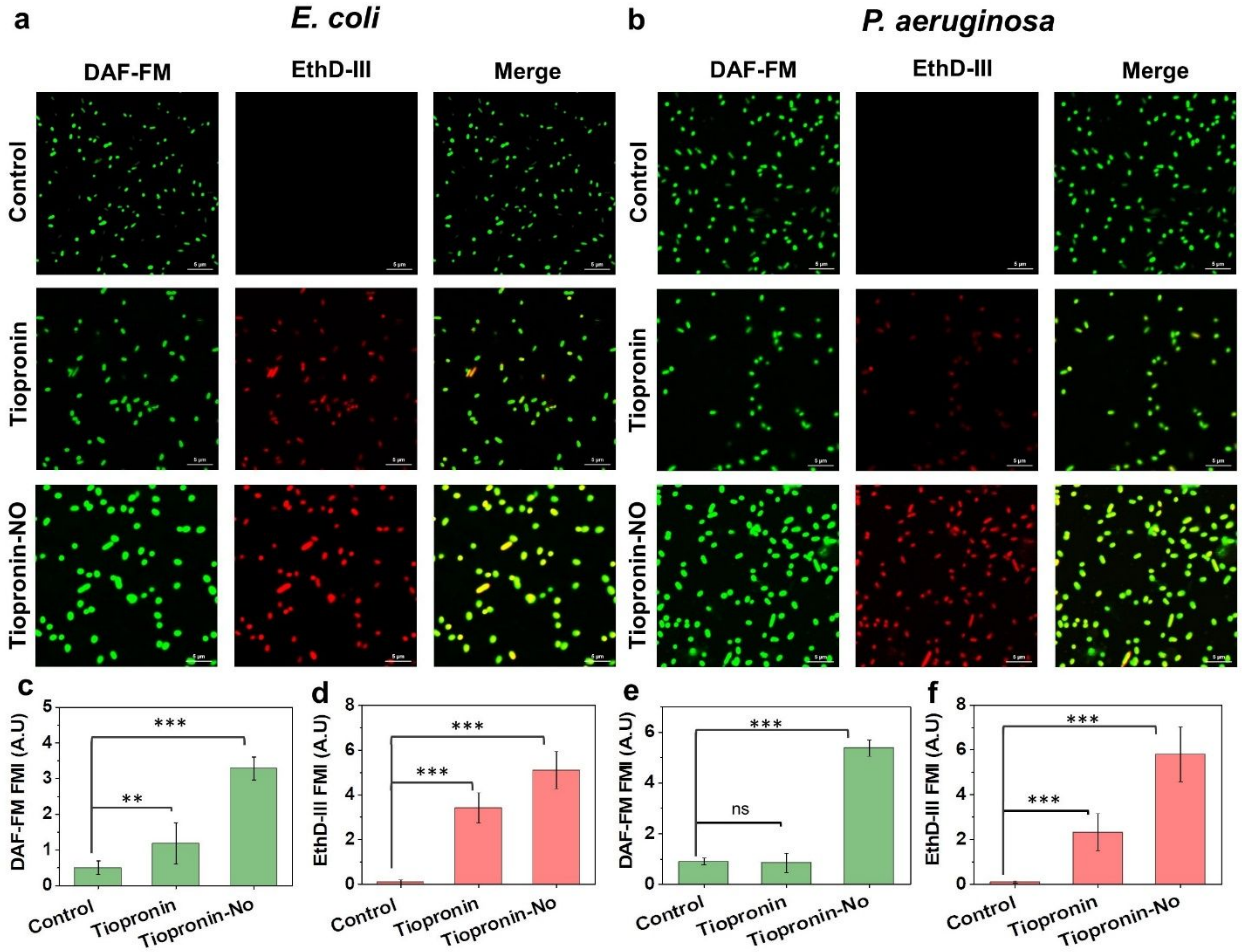

Figure S4. Localization of NO in bacterial cells and their impact in bacterial killing. (a) Live cell imaging results of intracellular NO labeling with *E. coli* and (b) *P. aeruginosa*. Both strains were treated with 10 mM of the drug for 24 h and cell imaging was performed using a confocal laser scanning microscope (scale bars = 5 μm), (c-f) Median fluorescence intensity (MFI) of DAF-FM, and EthD-III based on imaging results from (a) and (b). Statistical significance is denoted as *p < 0.05; **p < 0.01; ***p < 0.001; ns, no significant difference.

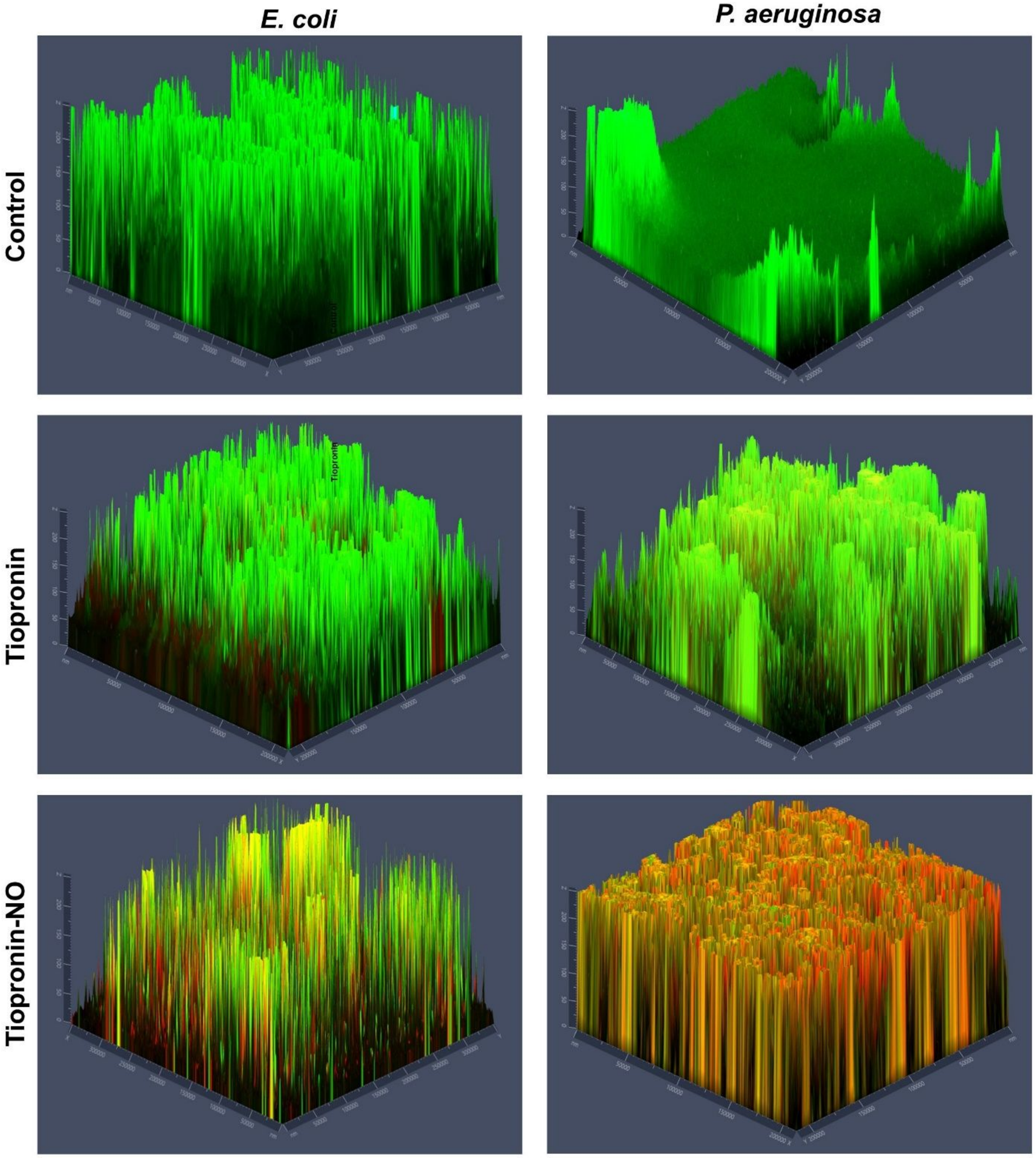

Figure S5. 3D image analysis of the treated biofilms based on imaging results from ***Figure 3.*** This image clearly shows the significant enhancement of the red signal, which indicates the number of dead cells present in the biofilm matrix after treatment with tiopronin and tiopronin-NO for 24 h.

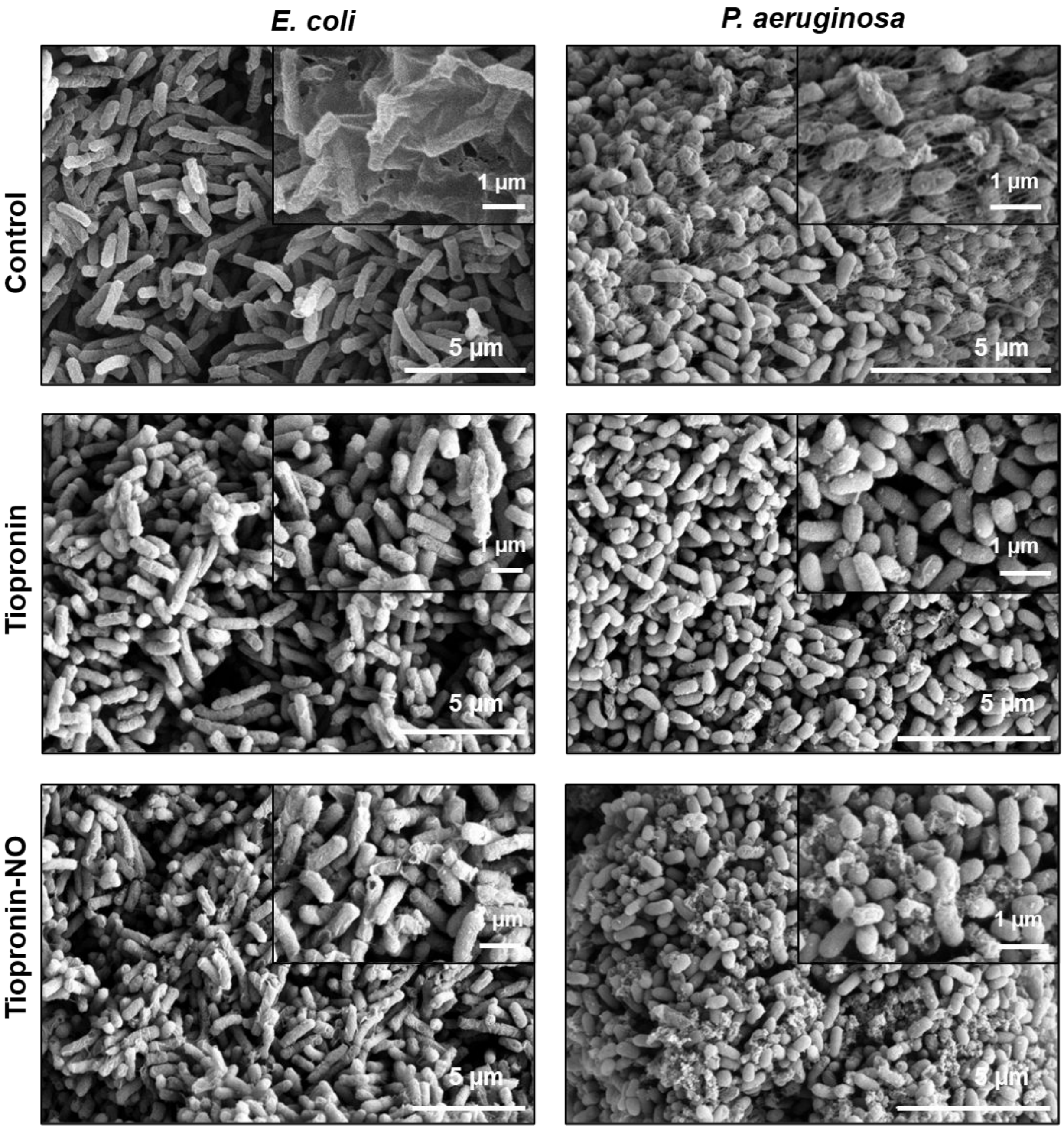

Figure S6. SEM images of *E. coli* and *P. aeruginosa* biofilms after treatment with tiopronin and tiopronin-NO for 24 h. Treatment with tiopronin-NO reveals bacteria with severely damaged and ruptured membranes accompanied by higher quantities of cell debris compared to control and tiopronin treatments. The scale bar represents 5 µm.

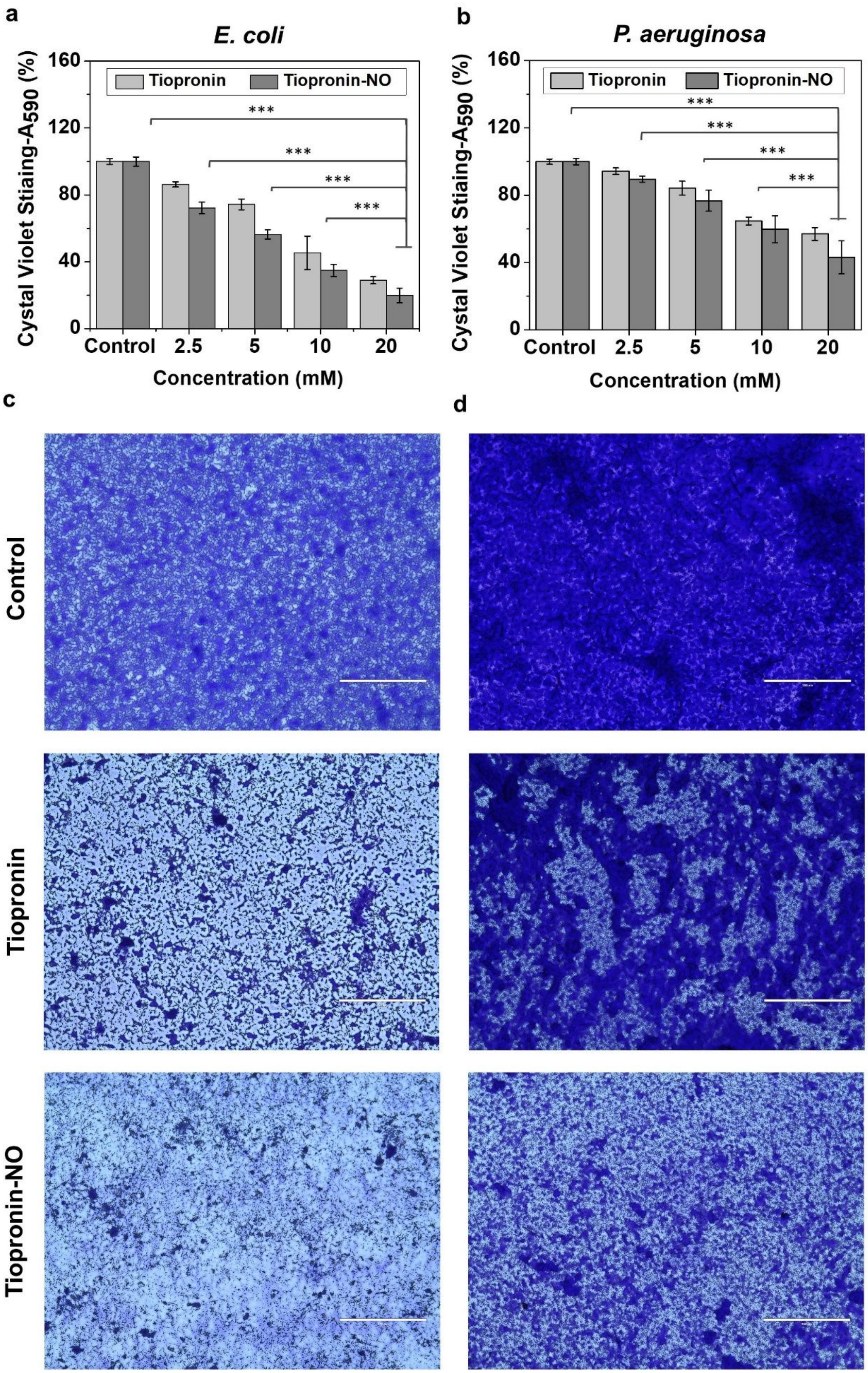

a
E. coli
Tiopronin
Tiopronin-NO
***
Cystal Violet Staing-A590 (%)
160
120
80
40
0
Control
2.5
5
10
20
Concentration (mM)
b
P. aeruginosa
Tiopronin
Tiopronin-NO
***
Cystal Violet Staing-A590 (%)
160
120
80
40
0
Control
2.5
5
10
20
Concentration (mM)
c
d
Control
Tiopronin
Tiopronin-NO

Figure S7. Inhibition of biofilm formation and quantification of biofilms after treatment with Tiopronin and Tiopronin-NO for 24 h and stained with crystal violet (CV) to assess biofilms formed by (a) *E. coli* and (b) *P. aeruginosa*. The solubilized cv was dissolved in 30 % acetic acid and optical density at $OD_{590\ nm}$ was measured for the quantification of biofilms. Typical bright field images of (c) *E. coli* and (d) *P. aeruginosa* biofilms stained with crystal violet after treatment with tiopronin and tiopronin-NO at a concentration of 20 mM for 24 h. The scale bar represents 200 μm. The values indicated are the average of three independent experiments (n = 5 for each strain). The results are shown as mean ± SD, and the asterisks represent a significant difference. *p < 0.05; **p < 0.01; ***p < 0.001; ns, no significant difference.

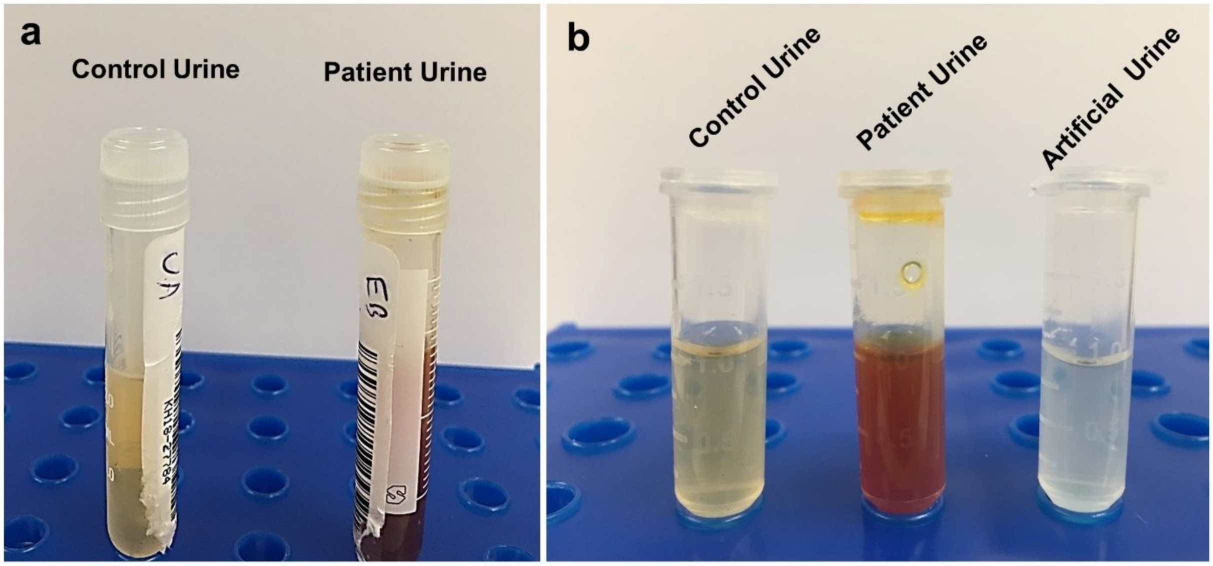

Figure S8. Urine samples. a) Images of the urine sample obtained from the vendor and used throughout the study. b) Image of the urine sample diluted 10 times in artificial urine. The patient urine sample contained hemoglobin, etc., and high amounts of protein were present in the sample from the patient with cystinuria (Details provided in Table S1).

Table S1. Details of urine samples

Sample	Hemoglobin	Protein	Glucose	Ketones	Bilirubin	Urobilinogen	Leukocytes /Erythrocytes	Nitrite	Bacteria
Control Urine	Neg (-)	Neg (-)	Neg (-)	3+(80 mg/dL)	Neg (-)	1mg/dL	Neg (-)	Neg (-)	Neg (-)
Patient Urine	3+ (Large)	3+(> 300 mg/dL)	100 mg/dL	2+(> 40-50 mg/dL)	Neg (-)	8mg/dL	Trace	Pos (+)	few

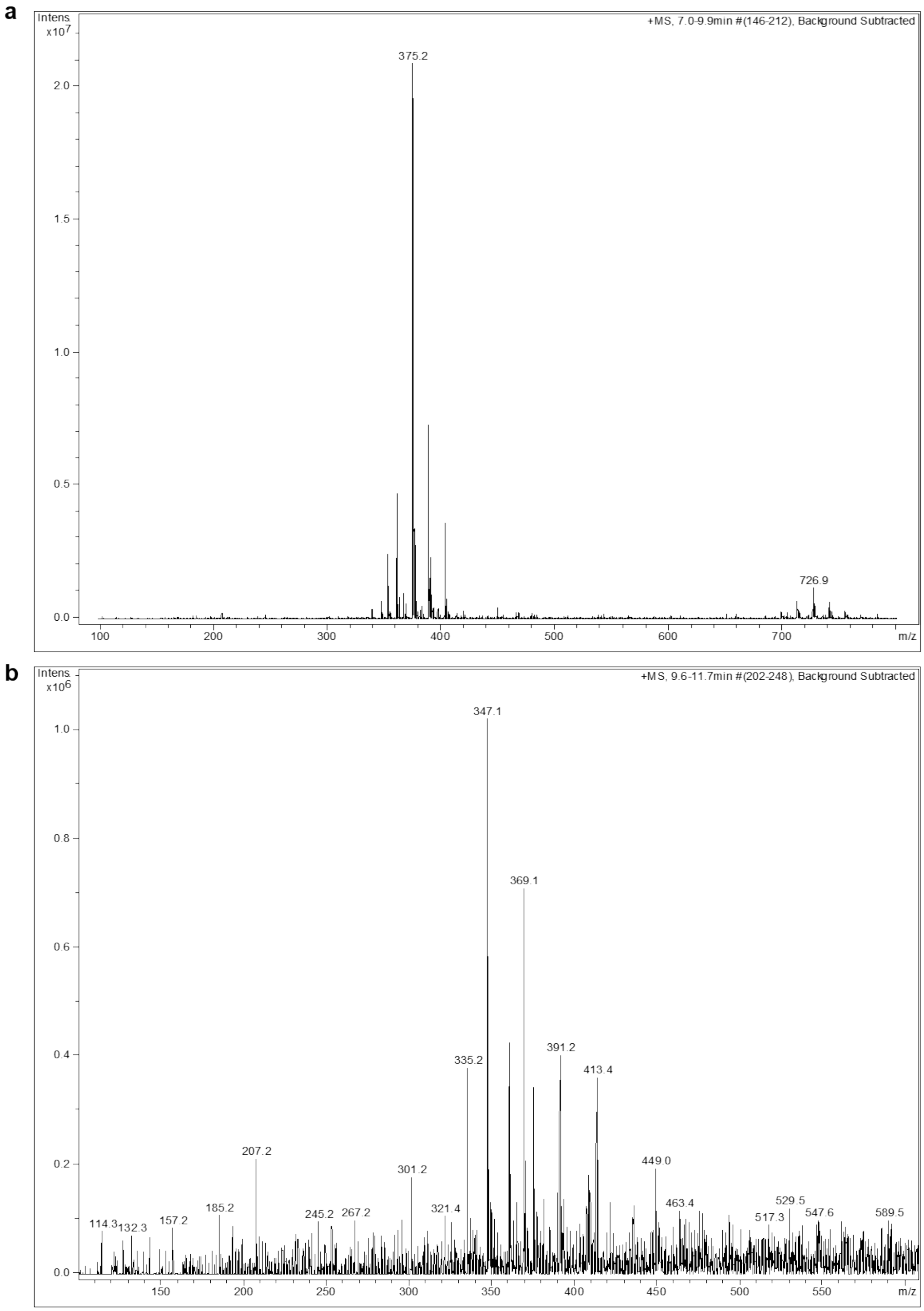

Figure S9. Mass analysis of urine sample treated with (a) tiopronin and (b) tiopronin-NO for 48 h at 37 °C.

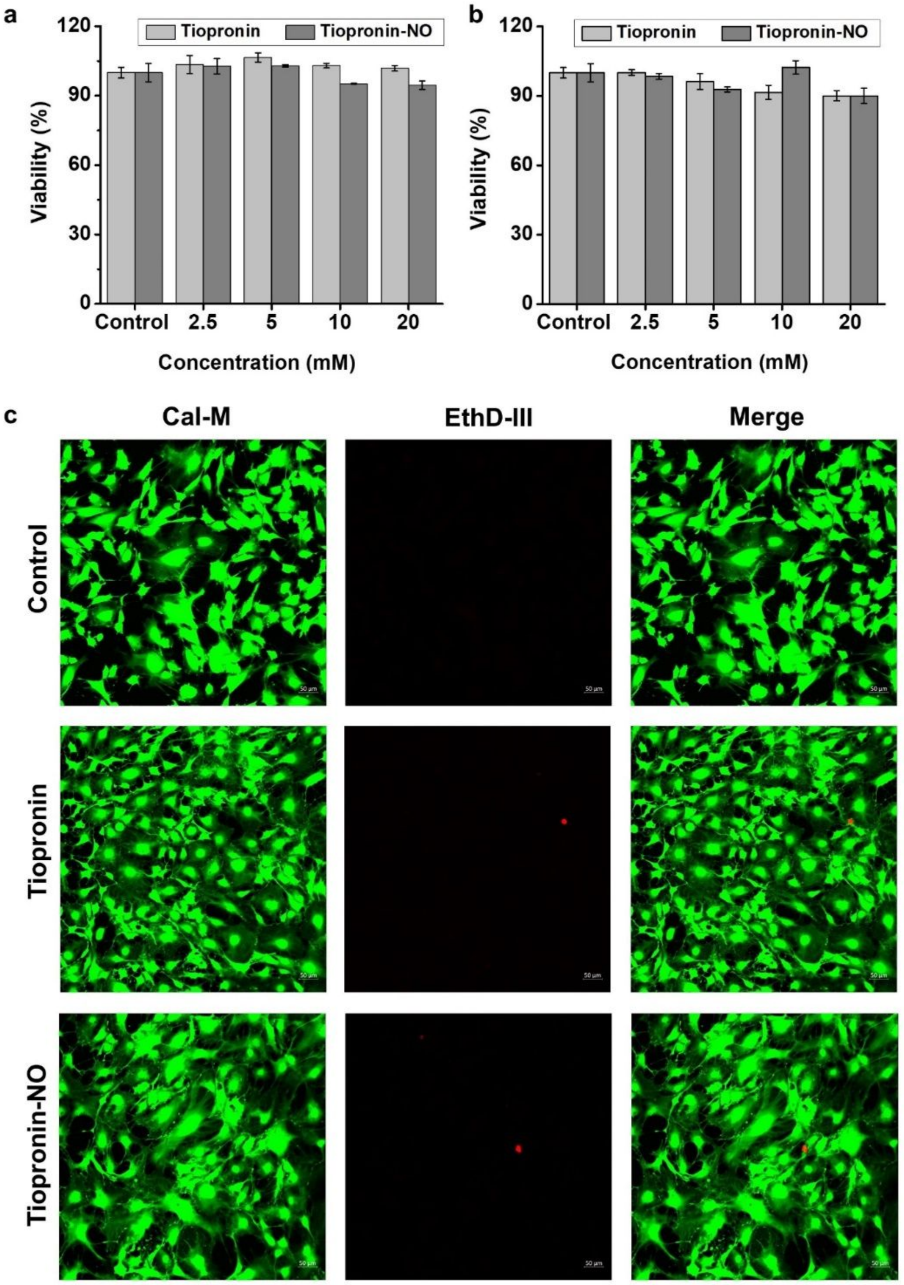

Figure S10. Effects of tiopronin and tiopronin-NO on mouse fibroblast cells at (a) 24 h and (b) 72 h after treatment with various concentrations. (c) Live/Dead cell imaging of the fibroblast cells treated with the drugs at a concentration of 20 mM for 24 h and imaging was performed using confocal laser scanning microscopy. The scale bar represents 100 µm. Data are expressed as the percentage of viability % ± SD of three independent experiments (n=5).

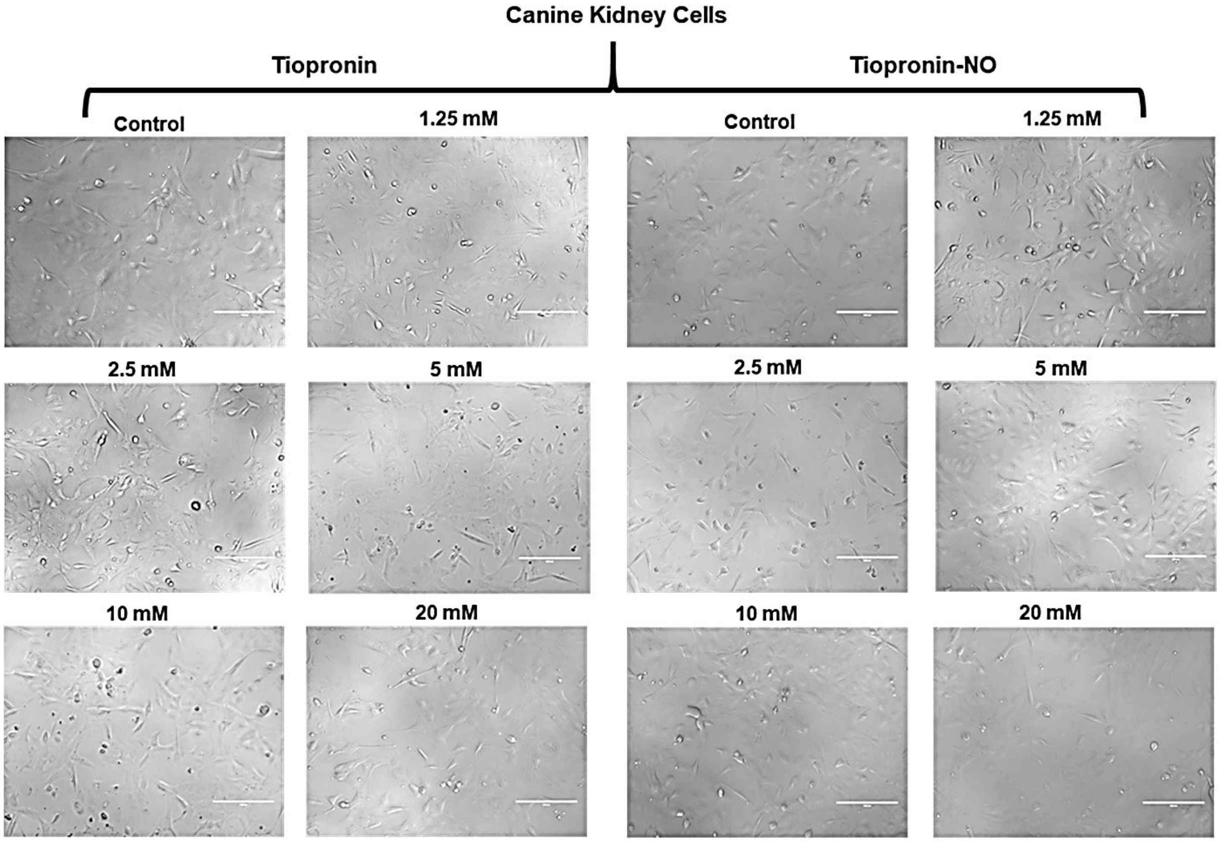

Figure S11. Effects of tiopronin and tiopronin-NO on canine kidney cells after treatment with various concentrations. Cells were imaged using bright field microscopy. The scale bar represents 200 μm.

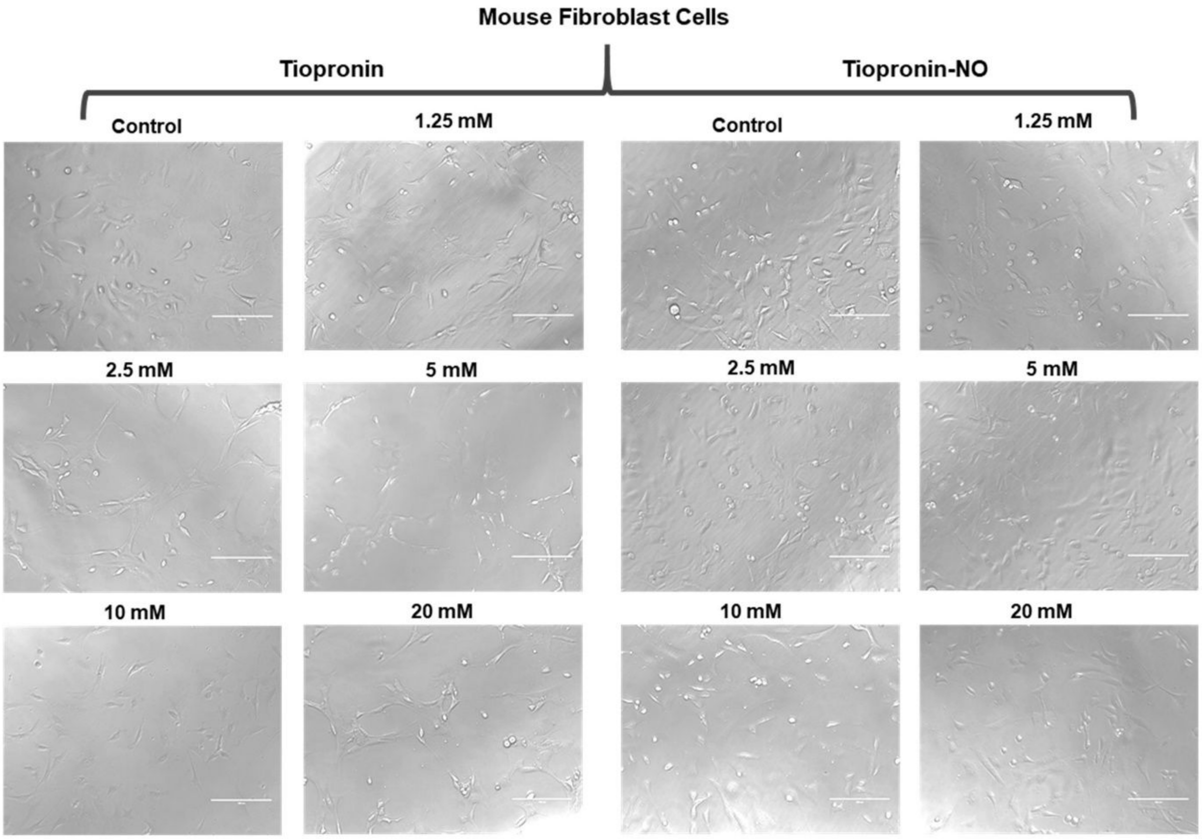

Figure S12. Effects of Tiopronin and Tiopronin-NO on mouse fibroblast cells after treatment with various concentrations. Cells were imaged using bright field microscopy. The scale bar represents 200 μm.
